# Exosome lncRNA IFNG-AS1 derived from mesenchymal stem cells of human adipose ameliorates neurogenesis and ASD-like behavior in BTBR mice

**DOI:** 10.1186/s12951-024-02338-2

**Published:** 2024-02-17

**Authors:** Yu Fu, Yuan-lin Zhang, Rong-qi Liu, Meng-meng Xu, Jun-ling Xie, Xing-liao Zhang, Guang-ming Xie, Yao-ting Han, Xin-Min Zhang, Wan-ting Zhang, Jing Zhang, Jun Zhang

**Affiliations:** 1 Research Center for Translational Medicine at East Hospital, School of Medicine, Tongji University, Shanghai, 200010 China; 2 Research Center for Translational Medicine at East Hospital, School of Life Science and Technology, Tongji University, Shanghai, 200010 China; 3 Key Laboratory of Spine and Spinal Cord Injury Repair and Regeneration of Ministry of Education, Orthopaedic Department of Tongji Hospital, School of Medicine, Tongji University, Shanghai, 200065 China; 4 Shanghai Institute of Stem Cell Research and Clinical Translation, Shanghai, 200092 China; 5 Department of Pathology, Air Force Medical Center, Beijing, 100142 China

**Keywords:** hADSC-Exos, hUCMSC-Exos, Neurogenesis, lncRNA IFNG-AS1, miR-21a-3p

## Abstract

**Background:**

The transplantation of exosomes derived from human adipose-derived mesenchymal stem cells (hADSCs) has emerged as a prospective cellular-free therapeutic intervention for the treatment of neurodevelopmental disorders (NDDs), as well as autism spectrum disorder (ASD). Nevertheless, the efficacy of hADSC exosome transplantation for ASD treatment remains to be verified, and the underlying mechanism of action remains unclear.

**Results:**

The exosomal long non-coding RNAs (lncRNAs) from hADSC and human umbilical cord mesenchymal stem cells (hUCMSC) were sequenced and 13,915 and 729 lncRNAs were obtained, respectively. The lncRNAs present in hADSC-Exos encompass those found in hUCMSC-Exos and are associated with neurogenesis. The biodistribution of hADSC-Exos in mouse brain ventricles and organoids was tracked, and the cellular uptake of hADSC-Exos was evaluated both in vivo and in vitro. hADSC-Exos promote neurogenesis in brain organoid and ameliorate social deficits in ASD mouse model BTBR T + tf/J (BTBR). Fluorescence in situ hybridization (FISH) confirmed lncRNA Ifngas1 significantly increased in the prefrontal cortex (PFC) of adult mice after hADSC-Exos intraventricular injection. The lncRNA Ifngas1 can act as a molecular sponge for miR-21a-3p to play a regulatory role and promote neurogenesis through the miR-21a-3p/PI3K/AKT axis.

**Conclusion:**

We demonstrated hADSC-Exos have the ability to confer neuroprotection through functional restoration, attenuation of neuroinflammation, inhibition of neuronal apoptosis, and promotion of neurogenesis both in vitro and in vivo. The hADSC-Exos-derived lncRNA IFNG-AS1 acts as a molecular sponge and facilitates neurogenesis via the miR-21a-3p/PI3K/AKT signaling pathway, thereby exerting a regulatory effect. Our findings suggest a potential therapeutic avenue for individuals with ASD.

**Graphical Abstract:**

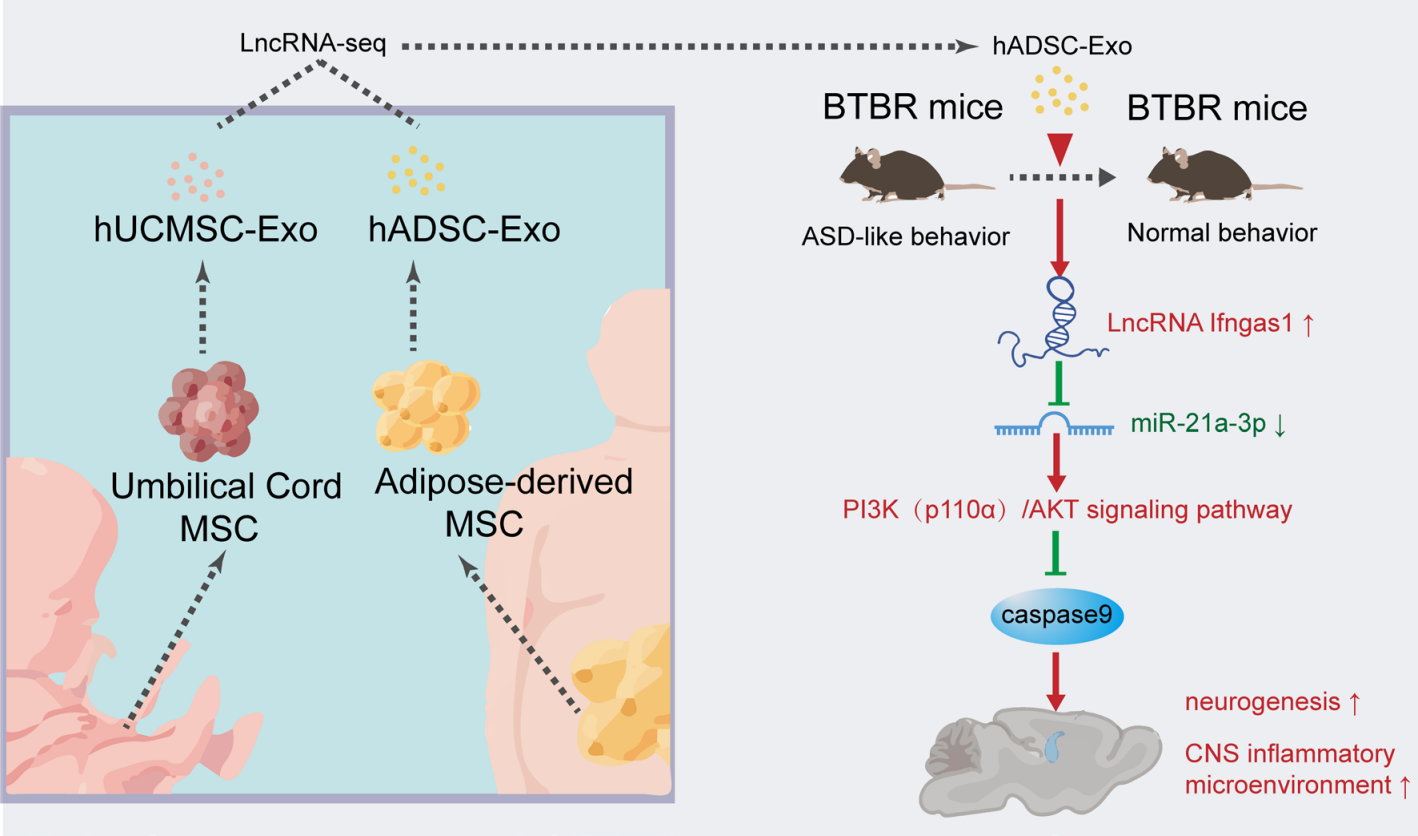

**Supplementary Information:**

The online version contains supplementary material available at 10.1186/s12951-024-02338-2.

## Introduction

Derived from adipose, umbilical cord and bone marrow tissues and organs, Mesenchymal stem cells (MSCs) are considered a prolific source for tissue engineering and regenerative medicine [1]. Exosomes, which range in size from 30–150 nm and contain a diverse array of proteins, mRNAs, long noncoding RNAs (lncRNAs), and other macromolecules [2]. Two distinct exosome types, hADSC-Exos and hUCMSC-Exos, offer numerous advantages over MSCs, including the retention of parent cell neuroprotection function, long-term stability, minimal immunological rejection, easily internalized into receptor cells, and lower probability of tumor development [3]. Consequently, hADSC-Exos and hUCMSC-Exos have emerged as a prospective cell-free treatment strategy for intervening in brain diseases [4]. Nevertheless, the heterogeneity in hADSC-Exos and hUCMSC-Exos remains unclear.

Exosomes contain lncRNAs, which have been shown to play a key part in regulating neurogenesis in individuals with ASD [5]. Through their ceRNA activity, lncRNAs can act as microRNAs (miRNAs) sponges and thereby contribute to endogenous neurogenesis, apoptosis, neural plasticity, and immune modulation [6–8]. A majority of ASD brains exhibit a common pattern of lncRNAs dysregulation, such as PTCHD1AS 1-3, SHANK2-AS and BDNF-AS [9]. Exosomes approximately reflect the intracellular status of their host cells, which implies their heterogeneity in different tissue source. However, the role of exosomal lncRNAs is inadequately understood in different tissue source.

The core symptoms of ASD are impaired social interaction, impaired communication, and repetitive stereotyped behavior disorder, a heterogeneous developmental disorder [10]. The potential neurobiological etiology of ASD includes GABAergic imbalances, impaired neurogenesis and neuroimmune processes [11]. The prevalence of ASD is increasing year by year, but currently, there is no established standard drug for patients with ASD [12]. Stem cell-based regenerative therapy has been widely concerned for their ability to treat diverse range of neurological disorders [13]. Recent studies have shown that transplantation of hematopoietic stem cells (HSCs) from the fetal liver is beneficial in alleviating ASD-like symptoms in children [14]. Transplantation of human amniotic epithelial cells (hAECs) corrects social deficits in BTBR mice, the specific mechanism of which is related to the promotion of hippocampal neurogenesis [15]. Despite the disclosure of the advantageous impact of MSC on the fundamental symptoms of BTBR mice, the precise mechanism through which MSC-Exo confers benefits to neurogenesis remains undisclosed [16].

In the present study, the lncRNA-seq of hADSC-Exos and hUCMSC-Exos to elucidate the functional diversity of MSC-Exos. Subsequently, the underlying mechanism responsible for the neuroprotective properties of hADSC-Exos was explored. In vitro experiments demonstrated that hADSC-Exos substantially enhanced the accumulation of neural progenitor cells (NPCs) and promoted neuron survival in brain organoids. In vivo, hADSC-Exos mitigated stereotyped and anxiety behavior, impaired new object recognition, and social deficits in BTBR mice. The administration of hADSC-Exos demonstrated the ability to ameliorate neurodevelopmental abnormalities and suppress the inflammatory microenvironment within the brains of BTBR mice. MiR-21a-3p expression was markedly upregulated in BTBR mice based on qRT-PCR results, which was effectively ameliorated by the intervention with hADSC-Exos. These findings indicate a potential regulator role of hADSC-Exos in the process of neurogenesis.

## Results

### Characterization of exosomes derived from hADSC and hUCMSC

**Fig. 1:**
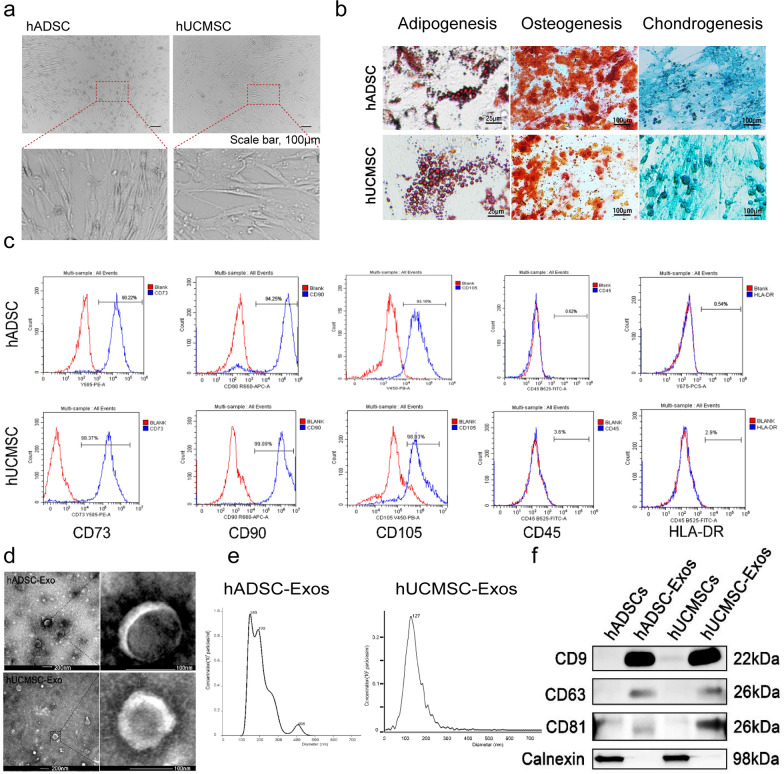
Identification of the exosome from hADSC and hUCMSC. **a** Morphology of hADSC and hUCMSC. Scale bar = 100 μm. **b** Multiple differentiation potential of hADSC and hUCMSC. Scale bar = 25 or 100 μm. **c** Surface markers profiling of hADSC and hUCMSC. The cells were highly positive for CD105, CD90, and CD73 and were negative for HLA-DR and CD45. **d** By TEM, purified hADSC-Exo and hUCMSC-Exo exhibit cup-like morphologies. **e** Nanoparticle analysis of hADSC-Exo and hUCMSC-Exo. **f** The protein markers in hADSC, hUCMSC, and their exosomes were analyzed by Western blot. hADSC-Exo and hUCMSC-Exo can express their common positive markers CD81, CD63, and CD9, however, the negative marker Calnexin is not expressed. WB analysis of whole cell lysates of the hADSCs and hUCMSCs shown in **f**

Human umbilical cord and adipose tissue samples were obtained as described in the “Materials and methods” section. Based on our published studies, hADSCs and hUCMSCs were cultured and their exosomes were isolated from their supernatant by differential ultracentrifugation, after getting enough exosomes, the exosomes were characterized and analyzed [17]. Typical morphology of hADSCs and hUCMSCs as indicated in Fig. 1a. Spindle-shaped morphology was observed in both hADSCs and hUCMSCs at 4–7 days after initial plating, and hADSCs and hUCMSCs at passage 5 (P5) observed in Fig. 1a both showed homogeneous fibroblastic-like morphology. According to Alcian blue staining, Oil red O staining, and Alizarin red S staining (ARS), our hADSCs and hUCMSCs can differentiate into Osteocytes, adipocytes and chondrocytes (Fig. 1b). The typical expression markers were identified by flow cytometry to determine the characteristics of mesenchymal stem cells [18]. Here, hADSCs and hUCMSCs showed a prominent expression of the mesenchymal stromal cell markers (CD73^+^, CD 90^+^, CD105^+^, CD45^−^ and HLA-DR^−^, Fig. 1c). We extracted exosomes by using hADSCs and hUCMSCs through ultrahigh-speed centrifugation (Additional file 1: Fig. S1). Transmission EM (TEM) revealed that exosomes from hADSCs and hUCMSCs had typical cup-shaped structures, with a diameter of around 100 nm (Fig. 1d). The exosome size was examined by Zetasizer Nano-Zs analyser, which revealed that the diameter of exosomes varies from 30 to 150 nm (Fig. 1e). Then, the well-known exosomal markers, CD9, CD81, CD63 and Calnexin were compared between hADSC-Exos and hUCMSC-Exos by western blot. CD9, CD81 and CD63 were both expressed in the hADSC-Exos and hUCMSC-Exos and did not show the expression of the endoplasmic reticulum protein Calnexin (Fig. 1f).

### LncRNAs sequencing analysis to identify hADSC-Exo and hUCMSC-Exo

**Fig. 2:**
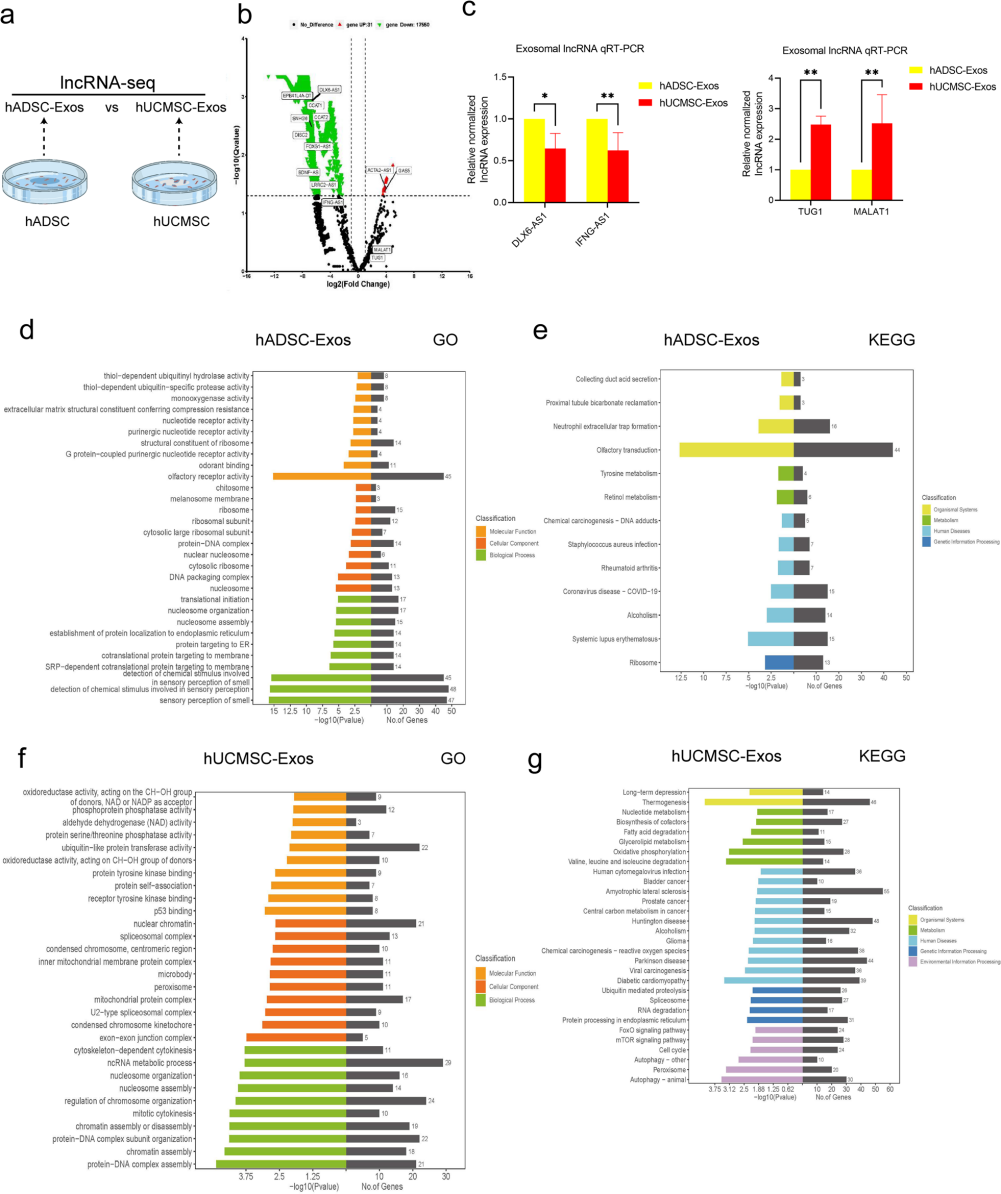
GO and KEGG analysis based on lncRNA-seq data of hADSC-Exo and hUCMSC-Exo. **a** Schematic of experimental design for experiments. Six samples proved for lncRNAs sequencing. Sample from hADSC-Exo2 contaminated sequencing batch were excluded. **b** Volcano diagram of differentially expressed lncRNAs. Red indicates higher-expressed lncRNA expression in hUCMSC-Exo, green indicates higher-expressed lncRNA expression in hADSC-Exo, and black indicates no difference in expression. **c** lncRNA sequencing data qRT-PCR validation. qRT-PCR validation of down-regulated candidate lncRNAs in hUCMSC-Exo (left). qRT-PCR validation of up-regulated candidate lncRNAs in hUCMSC-Exo (right). **d** GO analysis of hADSC-Exo lncRNAs targetgene. **e** KEGG pathway of hADSC-Exo lncRNAs targetgene. **f** GO analysis of hUCMSC-Exo lncRNAs targetgene. **g** KEGG pathway of hUCMSC-Exo lncRNAs targetgene

With the aim of exploring the similarities and differences between hADSC-Exo and hUCMSC-Exo, exosomal lncRNAs were sequenced in hADSC-Exo and hUCMSC-Exo (Fig. 2a and Additional file 1: Fig. S2a–d). When comparing hADSC-Exo to hUCMSC-Exo, BDNF-AS and other lncRNAs were higher expressed in hADSC-Exo. GAS5 and other lncRNAs were higher expressed in hUCMSC-Exo in volcano plot (Fig. 2b). Next, we used qRT‒PCR analysis to confirm the expression of several lncRNAs in hADSC-Exo to hUCMSC-Exo. The results showed that the expression of lnc-DLX6-AS1 and IFNG-AS1 were higher in hADSC-Exo compared with that of hUCMSC-Exo. The results showed that the expression of lnc-TUG1 and MALAT1 were higher in hUCMSC-Exo compared with that of hADSC-Exo (Fig. 2c). We carried out GO term enrichment and KEGG pathway enrichment analysis of target genes of lncRNAs in hADSC-Exo to elucidate the potential biological role of hADSC-Exo. The GO analysis revealed that the DEGs were significantly associated with nucleotide receptor activity and translational initiation (Fig. 2d). KEGG pathway analysis showed that rheumatoid arthritis, alcoholism and ribosomal are the main pathways among the upregulated gene (Fig. 2e). The hUCMSC-Exo lncRNAs targetgene GO analysis showed that the DEGs were notably engaged in aldehyde dehydrogenase (NAD) activity and ncRNA metabolic process (Fig. 2f). KEGG pathway analysis showed that Valine, leucine and isoleucine degradation and cell cycle are the main pathways among the upregulated gene (Fig. 2g). The above results suggest that hADSC-Exo protection in immune microenvironment. The pathogenesis of ASD including immune dysregulation, environmental factors, hereditary susceptibility, etc. Our subsequent experiments will explore hADSC-Exo's effects on neurodevelopment and its role in ASD, based on the high abundance and reproducibility of hADSC-Exo lncRNAs.

### hADSC-Exo suppress proliferation of NSC and tend to promote neurogenesis in brain organoids

**Fig. 3:**
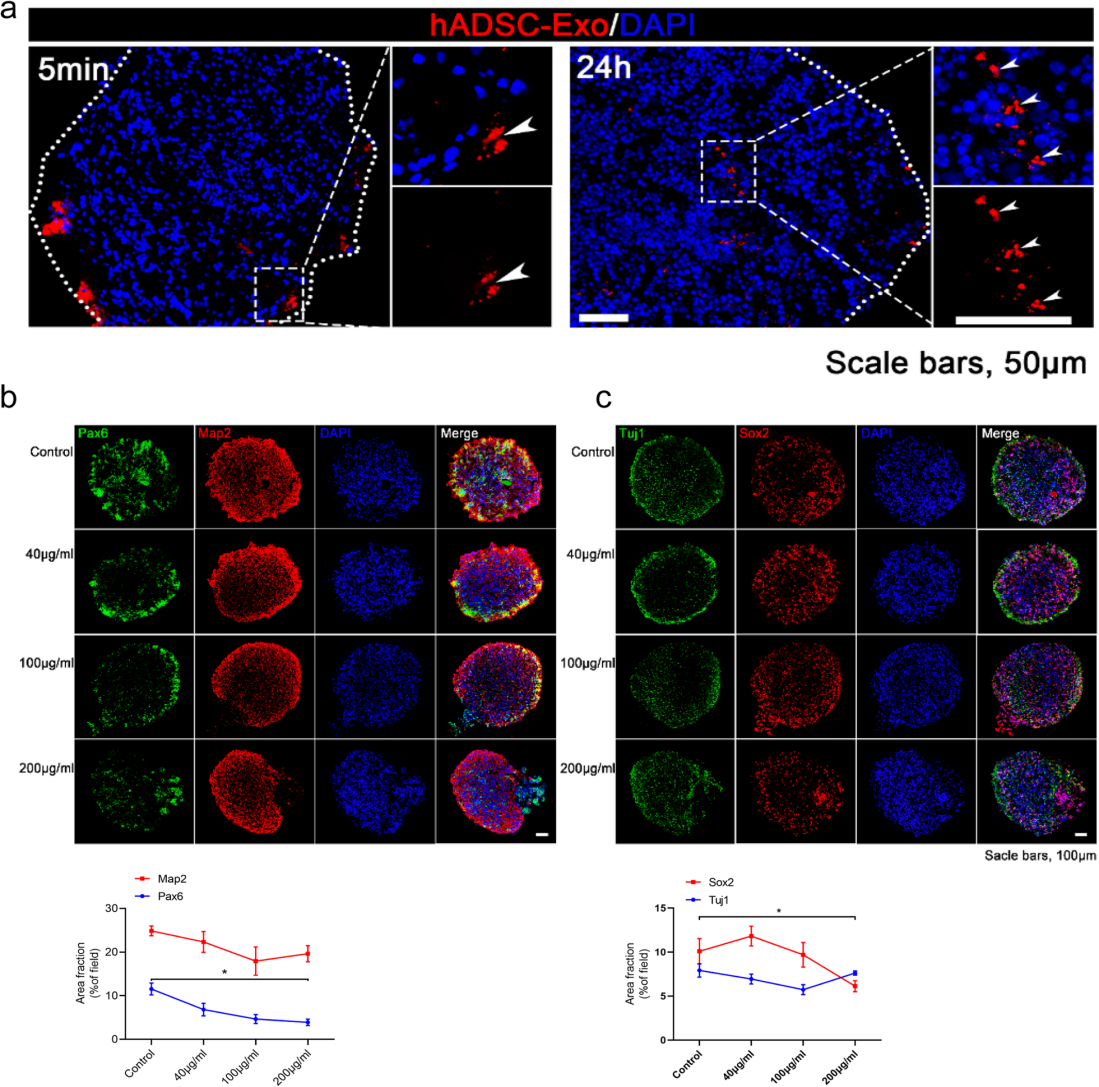
hADSC-Exo treatment inhibits the proliferation of NPC while showing beneficial effects of neurogenesis within brain organoids. **a** PKH26-labeled hADSCs-Exo (red) retention in the site of brain organoids. Scale bar = 50 μm. Nuclei (DAPI; blue). **b**, **c** Immunofluorescence double-staining of formalin-fixed brain organoids sections. **b** Immunofluorescence staining for Pax6 (red), Map2 (green), and DAPI staining (blue fluorescence). **c** Double immunofluorescence for Sox2 (green) and Tuj1 (red). A two-way ANOVA followed by Tukeys multiple comparison test was applied to all data. Error bars represent S.E.M. *p < 0.05, **p < 0.01, ***p < 0.001

To further determine the contribution of hADSC-Exo during neurodevelopment, a three-dimensional (3D) environment co-culture system was established. Hence, we generated brain organoids following a previous study's method [19]. Immunofluorescence revealed that PKH26-labeled hADSC-Exo has the capability to enter the brain organoids after 5 min co-culture. After 24 h, implying that hADSC-Exo might have entered the brain organoids (Fig. 3a). To determine the optimal concentration of hADSC-Exo, brain organoids were randomly assigned to four different concentration groups, with the no treatment control group and the other three groups applying 40 μg/mL, 100 μg/mL, 200 μg/mL of hADSC-Exo for one time two days. Diameters of organoids were quantified at 1 d, 12 d, 30 d after co-cultured. Morphological images intuitively revealed that the diameter of the organoid after co-culture with hADSC-Exo for 30d was lower compared to the control group (Additional file 1: Fig. S3a). The co-cultured brain organoids were immunostained for β-Tubulin (Tuj1), glial fibrillary acidic protein (GFAP), nucleus related antigen (Ki-67), Nestin, recombinant paired box gene 6 (PAX6), Sox2 to test whether the hADSC-Exo affected cell lineage choice (Fig. 3b, c and Additional file 1: Fig. S3b, c). Although there was no significant difference in the ADSC-Exo treated group, a downward trend in the numbers of astrocytes (GFAP^+^) and an upward trend in the number of neurons (Tuj1^+^) compared to the control group was observed (Additional file 1: Fig. S3b). Next, we investigated whether hADSC-Exo can affect the formation of immature and mature neurons. Immunocytochemistry was performed to assess neural progenitor cells (NPCs) proliferation using an NPC-specific marker, PAX6. MAP2, a marker of mature neurons [20]. The numbers of NPC (PAX6^+^) were markedly decreased in the hADSC-Exo treated group, whereas those of mature neurons (MAP2^+^) showed a trend to increase at 100 μg/mL concentration (Fig. 3b). Similarly, the numbers of NPC (SOX2^+^) were markedly decreased in the 200 μg/mL hADSC-Exo treated group (Fig. 3c). Those results suggest that hADSC-Exo induces depletion of NPC and promotion of its differentiation, may contribute to alleviating neurodevelopmental disease models. In addition, the next experiment was conducted with an intermediate concentration of 100 μg/mL.

### hADSC-Exo can regulate the expression of neurogenesis and brain inflammation-related genes in BTBR mice

**Fig. 4:**
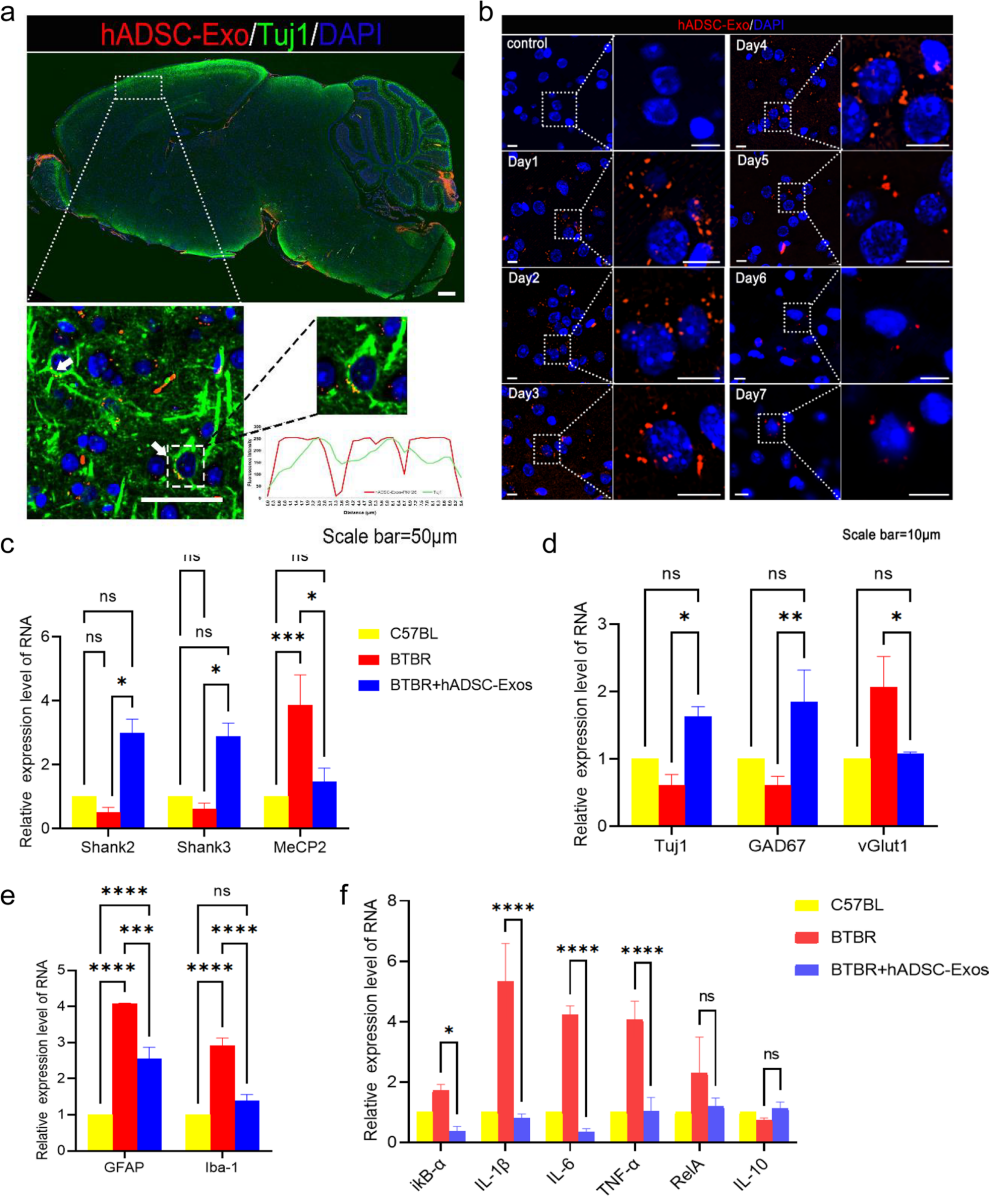
The hADSC-Exo treatment demonstrates the ability to modulate the expression of genes associated with neurogenesis and brain inflammation in BTBR mice. **a** Mouse brain localization injection of hADSC-Exos. PKH26-labeled hADSC-Exos co-localized with Tuj1 + neurons. **b** PKH26-labelled hADSC-Exos was localized and injected, and tissues were collected from day 1 to day 7 for immunofluorescence staining. **c**–**f** qRT-PCR. **c** ASD marker gene expression examined by qRT-PCR. **d** Neuron genes expression confirmed by qRT-PCR. **e** Astrocyte and microglia activation genes expression detected by qRT-PCR. **f** Inflammatory genes expression analyzed by qRT-PCR. All data were analyzed by two-way ANOVA followed by Tukeys multiple comparison test. Statistical differences were analyzed by two-way ANOVA followed by Tukeys multiple comparison test. Bars represent mean and error bars S.E.M. *p < 0.05, **p < 0.01, ***p < 0.001

BTBR mice (BTBR T + tf/J) are one of the most widely used ASD inbred strain mouse models (Additional file 1: Fig. S4a–c). Moreover, in the adult brains of BTBR mice, a reduced synaptogenesis was observed which is the critical step in neural network formation (Additional file 1: Fig. S4d). Studies have shown promising efficacy of human amniotic epithelial cells in improving BTBR mice behavior [15, 21]. However, the underlying mechanisms of hADSC-Exo’s protective role have not been elucidated. We asked whether hADSC-Exo promotes neurogenesis in BTBR mice. We were firstly labeled with the lipophilic dye PKH26 and then chose intracerebroventricular (ICV) infusion of hADSC-Exo. Before ICV, all the mice were confirmed to be in good physical condition with glossy hair and regular diets. Mice were adapted to the environment for 3 days. After ICV, the mice in the hADSC-Exos group and hADSC-Exos Prok group exhibited the same condition recorded prior to ICV. To investigate the location of hADSC-Exo in BTBR mice brains, we carried out FISH on adult mice brains. Colocalization of hADSC-Exo (red) with Tuj1 (green) revealed that hADSC-Exo can be measured in neurons of the cortex in BTBR mice (Fig. 4a). The retention time of hADSC-Exo in BTBR mice is d = 7 days long (Fig. 4b). The expression of ASD marker genes, such as *Shank2, Shank2, MeCP2* were examined in BTBR mice. RT-PCR revealed that ASD marker genes were reversed in BTBR mice after hADSC-Exo intervention, and tended to normalize compared with WT (Fig. 4c). The expression of neuron genes, such as *Tuj1, GAD67 (GABAergic neuron marker), vGLU1 (glutamatergic neuron marker)* were examined in BTBR mice (Fig. 4d). hADSC-Exo improves not only neurogenesis, but also the imbalance between excitatory and inhibitory neurotransmission in BTBR mice brain. Increased expression of *GFAP* and *Iba-1* are the most important markers of astrocyte and microglia activation. There was activation of microglia and astrocytes throughout the brain, resulting in inflammation-related factors in the brain. An elevated *GFAP* and *Iba-1* expression level, which is considered as the implicating intracerebral inflammation in BTBR mice brain (Fig. 4e). Inflammatory factor levels were detected using RT-PCR for ikB-α, IL-1β, TNF-α, and IL-6. The hADSC-Exo may effectively improve the intracerebral microenvironment and alleviate the inflammatory condition.

### hADSC-Exo treated ameliorate inflammation and promote neurogenesis in PFC regions of BTBR mice brain

**Fig. 5:**
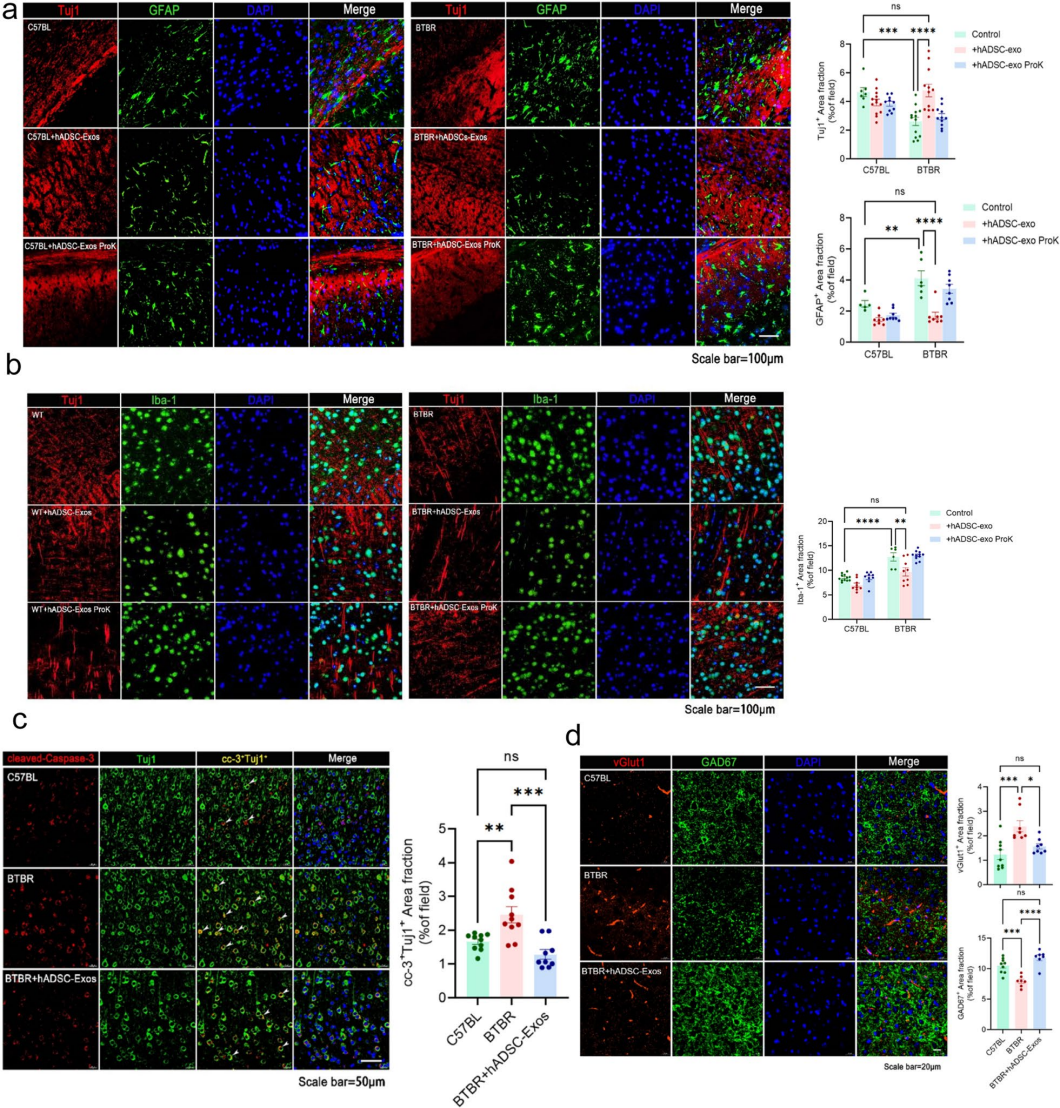
BTBR mice brains treated with hADSC-Exos showed reduced inflammation and increased neurogenesis in the PFC region. **a**–**d** Immunofluorescence double-staining of formalin-fixed mice brain sections. **a** Staining for committed neuronal markers GFAP (green immunofluorescence) and Tuj1 staining (red immunofluorescence) in the PFC is shown, and DAPI staining (blue fluorescence). **b** Iba-1 fluorescence in green and Tuj1 immunofluorescence in red (Texas Red), and DAPI staining (blue fluorescence). **c** Representative immunofluorescence images reveal Tuj1 (green) and CC-3 (red) immunoreactive (Tuj1 + /CC-3+) cells in the PFC region. **d** The red color is vGlut1 staining and the green color is GAD67, and nuclear staining by DAPI staining (blue fluorescence). A two-way ANOVA followed by Tukeys multiple comparison test was applied to all data. Error bars represent S.E.M. *p < 0.05, **p < 0.01, ***p < 0.001

To verify whether hADSC-Exo affects astrocyte and microglial cell activation in vivo, we examined the proportions of Tuj1^+^, GFAP^+^ cell and Iba-1^+^ cell in the PFC of mice by immunostaining. While neuron was significantly increased after hADSC-Exo treated, the astrocyte and microglial cell activation were significantly reduced. The percentage of neuron strikingly increased by twofold as compared with non-treated BTBR mice (*p* = 0.00259, Fig. 5a). While the increase in neuron was significant, GFAP^+^ cells dramatically decreased by 1.6-fold (*p* = 0.0048) and Iba-1^+^ cells by twofold in BTBR mice (*p* < 0.0001, Fig. 5a and b). We asked whether such a cell number increase could be attributed to a reduced apoptotic rate. Immunostaining of the PFC region of the brains of BTBR mice was analyzed for the apoptotic marker, cleaved Caspase-3 (CC-3) [22]. CC-3^+^cells levels decreased in PFC of BTBR mice after hADSC-Exo treated. The addition of hADSC-Exo was able to protect the cells apoptosis and remarkably reduce the rate of apoptotic cells. Meanwhile, vGlu1^+^ cells increased by 2.1-fold (*p* < 0.0001) and GAD67^+^ cells decreased by twofold (*p* = *0.0012*) in BTBR mice after hADSC-Exo treatment. Altogether, these results suggested that hADSC-Exo administration improved the CNS inflammatory microenvironment and promoted neurogenesis in PFC regions of BTBR mice brain.

### hADSC-Exo treated reverses ASD-like behavior in BTBR mice

**Fig. 6:**
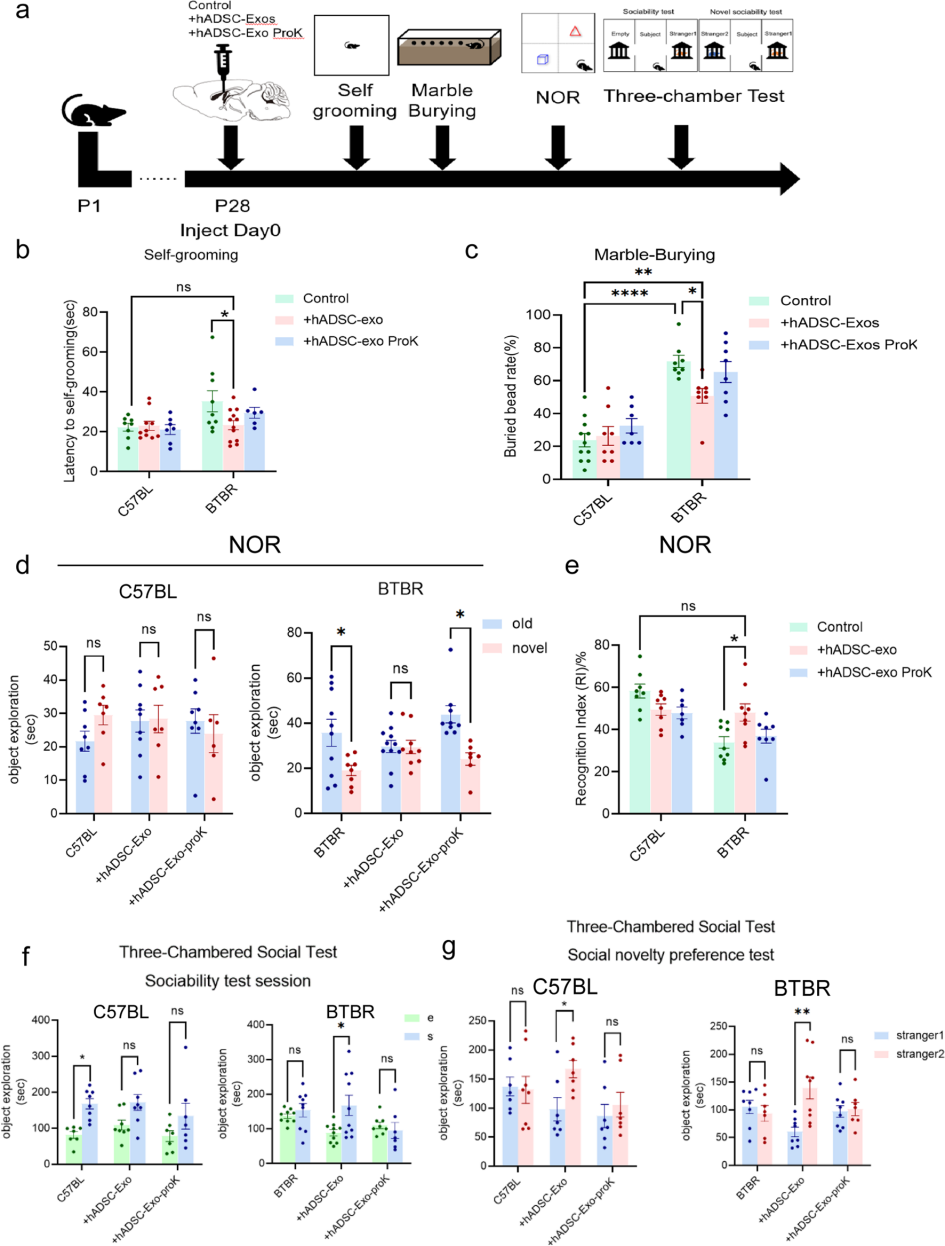
The administration of hADSC-Exos effectively ameliorates ASD-like behavior in BTBR mice. **a** Timeline of tests for extensive behaviors. **b** Self-grooming test. 10-min’ self-grooming test is conducted. **c** Marble burying test. 10-min’marble burying test is conducted. **d**, **e** Novel object preference (NOP) test. Each group was allowed to explore objects in order to test its novelty preference using the novel object preference test. **f**, **g** Three-chambered test. After 3-day training period in which mice were exposed to the unfamiliar mice #1, unfamiliar mice #2 was placed in sociability test session (**f**) and social novelty preference (**g**). Quantified the residence time of the experimental mice in the unfamiliar mice area. Two-way ANOVA with Tukey’s multiple comparison test. n = 6–12 mice per group for behavior, and Bars represent mean and error bars S.E.M. *p < 0.05, **p < 0.01, ***p < 0.001

To further validate the effects of hADSC-Exo on cortical neuronal function, we performed a series of ASD core behavioral tests on BTBR mice (Fig. 6a). Behavior tests were performed 2 weeks following ICV to determine the condition of mice. Proteinase K treated hADSC-Exos were used as negative control [23]. Firstly, we use self-grooming test to confirm whether hADSC-Exo treatment affects stereotyped repetition behavior. hADSC-Exo-treated BTBR mice displayed lower self-grooming time than BTBR mice and tended to normalize compared with C57BL WT mice (Fig. 6b,* p* = 0.0245). Similarly, in marble burying test, more marbles were buried by BTBR mice than C57BL WT mice. (*p* < 0.0001). The rescue effect was only observed in the BTBR mice, the hADSC-Exo-treated exhibited fewer marbles than the untreated BTBR mice (*p* = 0.04, Fig. 6c). In conclusion, these results indicate that the stereotyped repetitive behaviors in BTBR mice can be rescued by hADSC-Exo. The ASD individual usually shows atypical impaired visual spatial ability [24]. The new object recognition experiment can be used to evaluate such visual recognition memory [25]. We next evaluated novelty preference of hADSC-Exo-treated BTBR mice by novel-object recognition test and socialization by a three-chambered test. During the novel-object recognition test, BTBR mice exhibited a decreased ability to identify the novel object, as shown by a considerably reduced proportion of time with the novel object to time with the familiar object than was observed in C57BL WT mice (*p* = 0.039). The large differences in preference for the novel and old objects are no longer present in BTBR mice after hADSC-Exo administration, suggesting an increase in cognitive function (Fig. 6d and Additional file 1: Fig. S5a). Moreover, hADSC-Exo-treated mice exhibited a higher preference index (Time^new^/Time^new^ + Time^old^) compared to non-treated BTBR mice (Fig. 6e,* p* = 0.025). In the social ability test (three-chambered test), we found that C57BL WT mice have an obvious curiosity tendency towards stranger mice, while BTBR mice are not interested in interacting with stranger mice (Additional file 1: Fig. S5b). The sociability of BTBR mice can be rescued by hADSC-Exo (Fig. 6f,* p* = 0.024). BTBR mice treated with hADSC-Exo showed greater interest in interacting with unfamiliar mice #2 in the social novelty preference test (Fig. 6g,* p* = 0.001). These data indicated that hADSC-Exo may rescue autism-like behaviors in BTBR mice such as stereotyped behavior, decreased visual-recognition memory, decreased sociability and visual-recognition memory.

### The lncRNA IFNGAS1 has been identified as significantly associated with ASD in hADSC-Exo

**Fig. 7:**
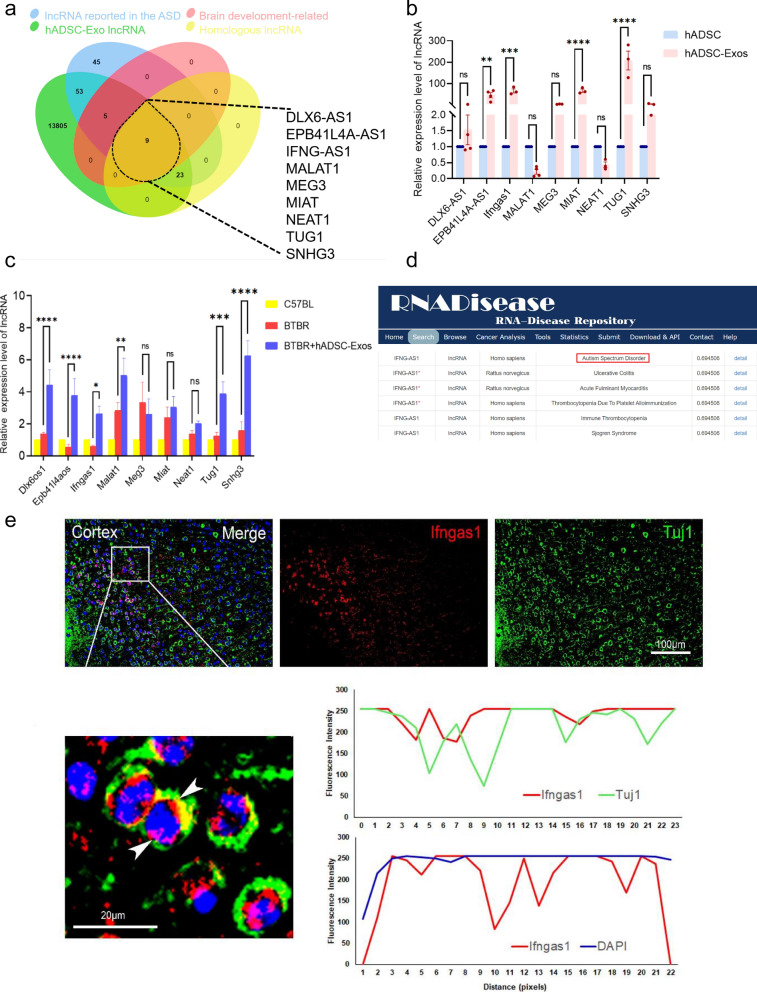
LncRNA IFNG-AS1 expression was significantly associated with ASD in hADSC-Exo. **a** Venn diagram demonstrating the intersections of genes from the ASD lncRNA set, brain development-related lncRNA set, hADSC-Exo lncRNA set and Homologous lncRNA data. **b** Quantification of the expression levels of nine candidate lncRNAs within hADSC-Exo was performed using qRT-PCR. **c** qRT-PCR technique was employed to detect the expression levels of 9 candidate lncRNAs in BTBR mice subsequent to treatment with hADSC-Exo. **d** Predicting on lncRNA Ifngas1-ASD associations in RNADisease database. **e** Combined immunofluorescence experiments of FISH with Tuj1 antibody (green) and Ifngas1 (red) show the distribution of Ifngas1 in neurons of cortical slices in WT mice. Enlarged images are shown at the bottom of the group. Scale bars = 100 μm (upper), 20 μm (lower). Two-way ANOVA as appropriate. By Tukeys multiple comparison test. Three biological replicates were used in the experiments. *p < 0.05, **p < 0.01, ***p < 0.001

To identify key genes in ASD, the centrally located hub lncRNAs in ASD-associated key modules were identified at first. ASD-associated lncRNAs obtained from the LncRNA Disease v2.0 (http://www.rnanut.net/lncrnadisease/), NONCODE (http://www.noncode.org/index.php), RNADisease (http://www.rnadisease.org/)、EVLncRNAs V2.0 (https://www.sdklab-biophysicsdzu.net/EVLncRNAs2/) [26–29]. The all lncRNAs were used as the final novel ASD-associated lncRNAs set, which finally resulted in a total of 117 lncRNAs. We also screened the lncRNAs homology between the human and mouse. By overlapping with these lncRNAs, we found that 9 candidate lncRNAs (Fig. 7a). They are DLX6-AS1 (Dlx6os1), EPB41L4A-AS1 (Epb41l4aos), IFNG-AS1 (Ifngas1), MALAT1 (Malat1), MEG3 (Meg3), MIAT (Miat), NEAT1 (Neat1), TUG1 (Tug1) and SNHG3 (Snhg3). The expression of 9 candidate lncRNAs in the hADSC-Exos was determined by qRT-PCR. In 9 candidate lncRNAs, 3 lncRNA-genes expressed higher, such as Epb41l4aos, Ifngas1 and MIAT (Fig. 7b). The expression level of 9 candidate lncRNAs was detected by qRT-PCR in BTBR mice after hADSC-Exos treated. The abnormal expression of lncRNA Ifngas1 and Epb41l4aos recover back to the normal status immediately after hADSC-Exos treated (Fig. 7c). It has been shown that the down-regulation of Epb4114aos (also known as EPB41L4A-AS1) leads to brain aging and neurodegenerative diseases by damaging the synthesis of NAD+ and ATP in the brain [30]. Studies on Epb4114aos have been conducted that are more relevant to cancer research [31]. Human lncRNA IFNG-AS1 (also known as NeST) shares homology with mouse ifngas1 and has two main functions. The development of regulatory T-cells is closely linked to Ifngas1 [32]. Regulatory T cell populations have been demonstrated to be critically involved in ASD. Studies reported the down-expression level of Ifngas1 in ASD patients' whole blood compared with healthy controls [33]. Ifngas1 was expressed in Tuj1^+^ neuron (green) and the presence of the protein in the different subcellular compartments was detected by FISH (Fig. 7d). This suggests that Ifngas1 expression may be associated with the neurogenesis. Ifngas1 was expressed in both the cytoplasm and nucleus (Fig. 7e). Therefore, Ifngas1 was identified as key lncRNA, which may have critical roles in ASD biological networks.

### Ifngas1 may act as a molecular sponge of miR-21a-3p, thereby negatively regulating Akt signaling pathway

**Fig. 8:**
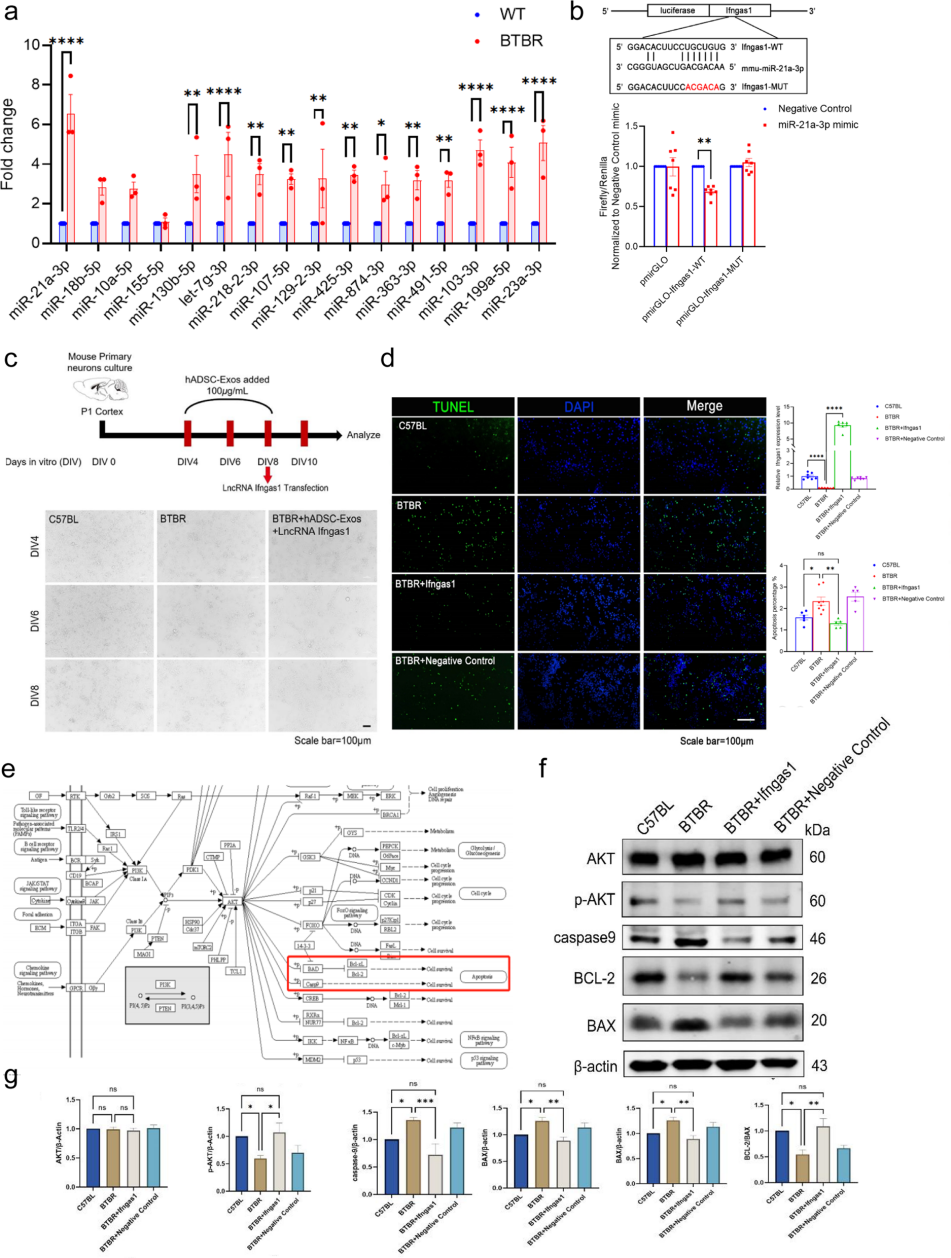
Ifngas1 acts as a molecular sponge regulating miR-21a-3p to down-regulated caspase9 expression, thereby regulating Akt signaling pathway. **a** qRT-PCR. Changes in the expression of miR-21a-3p, miR-18b-5p, miR-10a-5p, miR-130b-5p and other ASD-related miRNAs after hADSC-Exo treated. **b** Firefly luciferase activity was normalized to Renilla luciferase activity (Firefly/Renilla). In 293T cells, Ifngas1 3′-UTR pmirGLO plasmid with miR-21a-3p mimic/negative control or mutant Ifngas1 (pmirGLO-Ifngas1-MUT) 3′-UTR pmirGLO plasmid with miR-21a-3p mimic/negative control was co-transfected. **c** Primary cultures of C57BL mice or BTBR mice neurons. BTBR mice neurons co-cultured with hADSC-Exo were transfected with Ifngas1. **d** Apoptosis was measured by IHC staining of cleaved caspase-3 (CC-3) in C57BL (WT) and BTBR mice neurons. BTBR mice neurons were transfected with Ifngas1 or NC mimic (negative control). **e** KEGG pathway enrichment diagram. **f**, **g** WB western blot. **f** In the neurons of BTBR mice, caspase9 and BAX levels are increased, while BCL-2 levels are decreased (relative to β-actin), which was related to a decrease in AKT phosphorylation (p-AKT), but the total level of AKT was unchanged. Transfection of Ifngas1 but not NC mimics (negative control) rescued the cortical neurons in BTBR mice. Quantification of AKT, p-AKT, caspase9, BCL-2, BAX, normalized to β-actin levels, and values were plotted. Differences in six protein values were assessed by Tukey’s multiple comparison test one-way ANOVA, error bars represent S.E.M. *p < 0.05, **p < 0.01, ***p < 0.001

A lot of evidence shows that lncRNAs can be used as miRNAs sponge to regulate the expression of target mRNAs [6]. A majority of ASD brains exhibit a common pattern of miRNAs dysregulation, including miR-21-3p and miR-146a [7, 8]. Previous studies revealed that there were 58 miRNAs differentially expressed in ASD brain compared with those in the healthy brain [34]. We performed RT-PCR in BTBR mice further verify the expression of ASD-associated miRNAs. In contrast, miR-21a-3p expression varied significantly in the cortex of different mice. In the cortex of WT mice, its expression was sevenfold higher than that of BTBR mice (*p* < 0.0001, Fig. 8a). miR-21a-3p can activate Akt signaling pathway and promote cell proliferation [35]. To determine whether Ifngas1 functions as a molecular sponge for miR-21a-3p in vivo, the double luciferase test was carried out using 293 T cells. The pmirGLO luciferase reporter vector was constructed from 300 bp sequence that includes the 3'-UTR seed region of Ifngas1. Afterwards, miR-21a-3p mimic was co-transfected with pmirGLO luciferase reporter vector. The levels of Firefly and Renilla luciferase activity were observed, indicating that miR-21a-3p directly targeted Ifngas1 (Fig. 8b). However, the candidate lncRNA Epb4114aos is not the target gene of ASD-related miR-21a-3p (Additional file 1: Fig. S6). To investigate the functional effect of Ifngas1 on neurogenesis, we initially analyzed variations in the morphology of cortical neurons cultured from C57BL mice, BTBR mice and Ifngas1 overexpressed hADSC-Exo-treated BTBR mice (Fig. 8c). After Ifngas1 overexpressed hADSC-Exo-treated, cortical neurons cultured from BTBR mice demonstrated significantly enhanced axon growth compared to those cultured from C57BL mice. Apoptosis in BTBR mice cortical neurons were rescued by Ifngas1 (Fig. 8d). Since miR-21a-3p has been reported to be antiapoptotic through PI3K(p110α) /AKT signaling pathway, here, we investigated the effect of Ifngas1/miR-21a-3p axis on AKT, p-AKT, caspase9, BCL-2, BAX. WB showed that the caspase9 increase in BTBR mice was associated with AKT phosphorylation, and that AKT could directly regulate BCL-2 and BAX in the PI3K (p110α)/AKT signaling pathway, without changes in the total protein level of AKT. Apoptosis is inhibited by Bcl-2, whereas apoptosis is promoted by Bax. The apoptosis is enhanced by the reduced ratio of Bcl-2 to Bax. These results substantiate that hADSC-Exo treatment promoted BTBR mice brain functional recovery by Ifngas1/miR-21a-3p axis to suppress neuronal apoptosis.

## Discussion

Compared with other conventional synthesized drug, exosomes have emerged as a promising therapeutic option for neurological diseases due to their ability to cross the blood–brain barrier [36, 37]. For cellular therapy products to be safe and effective, comprehensive characterization, identification of the most relevant critical quality attributes (CQAs), and quality control are necessary. Researchers have devoted substantial efforts to elucidate the biological properties of exosomes and their components and their roles in central nervous system disease, such as AD, Parkinson's disease (PD) and Huntington's disease (HD) [38]. At the same time, several exosome databases have been created. ExoRBase 2.0 database have shown that exosomes in the human biofluids also contain different lncRNA [39]. LncExpDB documents exosomal lncRNAs differentially expressed across diverse biological conditions [40]. EVAtlas houses the expression profiles of ncRNA types in EV samples from human tissues, but not yet for lncRNAs [41]. Exosomes may carry specific lncRNAs and miRNAs from a variety of tissue sources [42]. Understanding the origin of exosomes is essential to gain insight into the prospective applications of human exosomes in neurotherapeutics [43]. However, intervention studies to date on the ASD have not fully paid attention to the origin of exosomes. Here, the two types of widely clinical used hMSCs and their exosome were obtained, and the lncRNA-seq of hADSC-Exo and hUCMSC-Exo was performed. Due to the key words “Olfactory transduction”, “Alcoholism” and “*Staphylococcus aureus* infection” in hADSC-Exo lncRNA-related KEGG analysis, we were reminded of its nervous-immune regulation ability (Fig. 2e). Hence, hADSC-Exo was selected to continue the study. Notably, there were only two biological replicates in hADSC-Exo. One hADSC-Exo 2 sample was excluded from the analysis since it might be contaminated. The Veen analysis shows similar patterns for hADSC-Exo1 and 3 samples (Additional file 1: Fig. S2c and d). LncRNAs intersect in three hUCMSC-Exo samples only 67 lncRNAs (Additional file 1: Fig. S2e). We used the union to do the comparison showed all differentially expressed lncRNA detected in hADSC-Exo and hUCMSC-Exo (Additional file 1: Fig. S2f). This prompts future research with larger samples of MSC-Exo, as the main limitation of our study is the low number of included hADSC-Exo and hUCMSC-Exo biological duplication. Verification of sequencing data through qRT-PCR is a key step in lncRNA-seq analyses. Among them, some lncRNAs were randomly selected for qRT-PCR, and the qRT-PCR results were consistent with the sequencing data, such as DLX6-AS1 and IFNG-AS1 (Fig. 2c).

Recently, Samir EL Andaloussi et al. investigated the effects of different exosome sources and doses on recipient cells in a systematic manner. They found that a low dose of exosomes produces profound transcriptional changes specific to the exosome cell source, while a high dose of exosomes produces a standardized response. High doses would likely overload the endocytic machinery and do not represent physiological conditions. Low doses would contain the fewest exosome-derived transcripts may not be able to be treated [44]. Their study highlights that standardization and comparable dosages should become common practice in exosome studies. Here, an analysis of bioinformatics-based cell sources was conducted, and doses were selected based on human brain organoids. Hence, we decided to choose an intermediate concentration. Our current study provides support for the standardization and comparable dosages of exosome paradigm to conducting initial clinical research.

The ASD mouse model BTBR mouse model recapitulates many features of the human ASD phenotype, such as GABAergic imbalances. Functional analyses show that the genes differentially expressed in the cerebral cortex of BTBR mice are mainly involved in the following biological processes: "neurological development", "social behavior", etc. [45]. The results of this analysis further demonstrated the similarity between the BTBR mouse model and ASD patients. Notably, in this study, we innovatively explored the efficient of hADSC-Exo in human brain organoid. Findings from human brain organoid and ASD mouse model provide the foundation for subsequent investigations in more complex systems. Due to the lack of approved drugs to treat the symptoms of ASD [5, 46], hADSC-Exo might be a good therapeutic agent in future.

It remains unclear how MSC-Exo contributes to neurogenesis in ASD, despite previous studies investigating its role in the disorder [5, 47, 48]. Elucidation of the mechanisms by which exosomal lncRNA ameliorate ASD-like behavior and neurogenesis is essential for intervention effectiveness. According to Joerger-Messerli et al., EVs derived from human Wharton's jelly MSC (hWJ-MSC)-MSCs may prevent and resolve HI-induced apoptosis in neurons in the neonatal brain by transferring let-7-5p from the EVs [49]. In this study, we illustrated that hADSC-Exos activated the PI3K(p110α)/AKT signaling pathway through the Ifngas1/miR-21a-3p axis. It can ameliorate neurogenesis and ASD-like behavior in BTBR mice. Exosomes contain lncRNAs and many other macromolecules from their source cells, such as proteins, miRNAs, etc. Further investigation of hADSC-Exos-mediated neuroprotection and neurogenesis mechanisms will be needed before considering ASD-related clinical practice. Moreover, it may be worth to test MSC-exo effect in ASD by other high-throughput approaches, including LC–MS proteome analysis and single cell sequencing as well. Overall, our findings add further insights into the hADSC-Exo functions in ASD.

## Materials and methods

### Ethics statements

All procedures followed the guidelines of the National Health and Medical Research Council of China and received approval from the Animal Ethics Review Committee of Tongji University.

### Cell culture

A healthy donor provided written informed consent to the East Hospital Affiliated to Tongji University to obtain the human adipose tissue samples. The ADSC cells in this study were utilized and the cells were serviced under the same conditions as those described in our previous study [17]. Human umbilical cord tissue samples were acquired from the Stem Cell Bank of the East Hospital Affiliated to Tongji University. After obtaining informed consent from the mother and her family, the donor signed a written informed statement. The approval was obtained from the East Hospital Ethical Review Board. All cells were incubated at 37 °C in an incubator with 5% CO2.

### Exo isolation and characterization

Exosomes isolated from the cell supernatant of hADSC/hUCMSC in the same methodology as described previously [50]. The cells were separated from the culture medium by centrifugation at 300*g* for 10 min. Transfer the supernatant to the new centrifugal tube and centrifuge for 10 min at 2000*g*, followed by an additional 30 min at 10,000*g*. The liquid supernatant was then centrifuged at 1,000,000*g* for 2 h at 4 °C (Additional file 1: Fig. S1). Afterward, exosomes were suspended in 1 × PBS and kept at − 80 °C for subsequent experiments.

### Exo characterization

The protein in exosome was measured to quantify exosomes. The concentration of Exo was evaluated using the bicinchoninic acid (BCA, Thermo Fisher Scientific, Waltham, MA, USA). In Fig. 1f, hADSCs and hUCMSCs samples of whole-cell lysate (WCL) was reserved. The WCL were boiled with SDS–PAGE sample loading buffer, separated by SDS–PAGE, blotted on PVDF membranes. Western Blot with anti-CD9, anti-63 and anti-CD81 is used for identifying exosome. Transmission electron microscopy (TEM, FEI, USA) was used to examine the morphology of the isolated exosomes [51]. Nanosight tracking analysis (NTA, Malvern, USA) was performed to analyze exosome morphology. In order to analyze particle numbers, the Nanoparticle Tracking Analysis (NTA) 3.0 software was used. hADSC-Exo was labeled with PKH26 using the PKH26 Red Fluorescent Cell Linker Mini Kit (Sigma, St Louis, MO, USA). Proteinase K used for chemical disruption of hADSC-Exo (Sigma, St Louis, MO, USA).

### Exo lncRNA-seq

The total RNAs were enriched from exosomes of hADSC and hUCMSC were extracted by RiboBio Co., Ltd, Guangzhou, China. Six RNA samples (hADSC-Exo and hUCMSC-Exo) were used in the subsequent analysis. Contaminated sequencing batch of hADSC-Exo2 samples were excluded. Normalized expression levels of the genes between the hADSC-Exo1 and 3 are well correlated with the Pearson correlation coefficient (R) values more than 0.99 (Additional file 1: Fig. S2b and c). In this paper, the differentially expressed lncRNAs were determined by |log2(FoldChange)|> 1 and Qvalue < 0.05, with thresholds for up- and down-regulated lncRNAs.

### Generation of cerebral organoids

The H9 human embryonic stem cell (H9 ES cell) used in this study was were donated by Professor Ru Zhang from Tongji University. H9 ES cells were cultured on Vitronectin XF™ (stem cell technologies, Canada) with mTeSR™ medium (stem cell technologies, Canada). After getting enough high-quality and low-differentiated H9 ES cells, then using the STEMdiff Cerebral Organoid kit (stem cell technologies, Canada). Briefly, on day 0, H9 ES cells were dissociated into single cells with ACCUTASE™ (stem cell technologies, Canada) and then suspended in embryoid body (EB) Seeding Medium. Every EB was embedded in 15 *µ*L of Cultrex UltiMatrix (R&D SYSTEMS, USA) and transferred into 6-well ultra-low attachment plate containing 3 mL expansion medium. After 10 d, embedded organoids' culture solution was replaced with maturation medium and organoids were placed on orbital shaker in 37℃ incubators at the speed of 65 rpm until day 50 [19]. For exosome co-cultured, based on the reference dose in literature, a concentration gradient is established to determine the optimal concentration [52, 53].

### Mice

Adult pairs of BTBR mice were acquired from The Jackson Laboratory (Bar Harbor, Maine) and were subsequently bred. The mice were housed in an SPF animal facility, where they were kept in white plastic enclosures with unlimited access to water and food.

### Exo transplantation

At the age of 4 weeks, BTBR male mice were fixed in a stereotactic frame (Ruiwode, Shenzhen, Guangdong Province, China). In the presence of 4% isoflurane, hADSC-exo, 2 μl per injection site, were injected into the cerebral lateral ventricles bilaterally at 0.5 *μ*L/min (Hamilton 701N syringe) to the following coordinates (relative to the bregma): anterior–posterior, − 1 mm; medial–lateral, ± 0.8 mm; dorsal–ventral, − 1.5 mm. Ten minutes after the needle was inserted, it was withdrawn. Animals were also treated with 0.3% gentamicin for 3 days around transplantation in order to suppress any possible immune response [54]. The behavioral experiment was done 2 weeks after the last treatment.

### Behavioral studies

Repetitive behavior was analyzed by a self-grooming test, in short, the mouse were kept in an empty cage (30 × 30 × 29.5 cm) for 10 min, during which time their self-grooming behavior was recorded [42].

Anxiety-like behavior was analyzed by marble-burying test, in short, twenty clean marbles (d = 14 mm) were evenly distributed on the surface of the corncob cushion (29 × 18 × 13.5 cm) at a depth of 5 cm. After 30 min of acclimatization in the test room, the mice were then placed in the test cage containing the marbles. The marbles were counted after 30 min of exploration [55].

The New Object Recognition test was executed in the opaque walled box (30 × 30 × 29.5 cm). The mouse was given 10 min to explore the arena without any objects before the adaptive test. Then, two familiar objects were fixed in the box and the mouse could explore two familiar objects for 10 min during the adaptive session. In the test session, a familiar object was changed by a new one, the mouse could explore all the objects for 10 min. The time spent by the mice exploring the novel object was analyzed and recorded. Recognition index = novel object recognition time/total object recognition time [56].

As previously described, the three-chamber test was employed to measure sociability behaviors [57]. In essence, the apparatus comprised three interconnected chambers with entryways. The mice explored the three-chambered apparatus for 10 min as part of the adaptation task. Afterward, the mice were allowed to interact either with an empty wire mesh cylinder or an unfamiliar mice#1 called stranger1. Mice were then able to explore the three rooms freely for 10 min. Following that, another mouse, unfamiliar mice#2 were placed in a opposite chamber, which was empty in the previous session, for the social novelty preference test. Finally, the time spent in the different chamber was counted.

### Immunohistochemistry (IHC)

Histological examinations were conducted on three mice from each group at random, IHC assay was performed as previously described [58]. Anti-ki67 (1:200, Abcam, UK, ab16667), anti-GFAP (1:1000, Abcam, UK, ab7260), anti-cleaved-caspase-3 antibody (1:500, Servicebio, GB11532), anti-Tuj1 (1:1000, Abcam, UK, ab7751) anti-Nestin (1:2000, Abcam, UK, ab221660), anti-vGlut1 (1:100, Biolegend, MMS-5245), anti-Iba-1 (1:500, Servicebio, GB11105), anti-GAD67 (1:500, Abcam, UK, ab13508). The aforementioned primary antibodies and secondary antibodies were used.

### Histology

After BTBR mice were euthanized, the hearts, lungs, kidneys, testes, brains, livers and spleens were dissected. The HE staining was carried out on tissue samples from at least three mice of each genotype [59]. Nissl staining was performed by incubating slides with 0.1% Nissl dye for 10 min [60]. To quantify hippocampal neurons, Golgi-Cox staining was used. The slides were first immersed in 1:1 hydrochloric acid and 100% ethanol for 2 h, then washed under running water [61].

### Dual-luciferase reporter gene assay

To generate the miR-21a-3p target site-containing 3′-UTR sequence of lncRNA Ifngas1, the overexpressing plasmid was used. A 1.5% agarose gel was used to purify the DNA fragments. Using XbaI enzyme-digested vectors pGL3-Control (Promega, Madison, WI, USA) to insert downstream of the luciferase gene [62]. The 293T cells, at 80–90% confluence, were co-transfected with lncRNA Ifngas1 3′-UTR and miR-21a-3p mimic. In vitro transfection was carried out with Xfect Transfection Reagent (Takara Bio, USA). In vivo transfection was performed using in vivo-jetPEI® reagent (Polyplus-transfection SA, France).

### Real-time PCR (qRT-PCR)

A MiRNeasy Mini Kit (Qiagen, Hilden, Germany) was used to extract miRNA and total RNA from tissues and cells. SYBR® Premix Ex TaqTM II Kit (RR820A, Takara Bio, Japan) was used to perform qRT-PCR. Primers were listed in Table 1 (Sangon Biotech, Shanghai, China). The quantitative PCR analysis was performed using an ABI7500 instrument (ABI Company, Oyster Bay, NY, USA).

**Table 1:** Primers information

Gene name	Gene type	Experiment	Primer (5′-3′)
*Gapdh*	Total RNA	qRT-PCR Forward	GCTGTCAACGATACGCTACGTAACG
		qRT-PCR Reverse	TGAAGGGGTCGTTGATCG
*Map2*	Total RNA	qRT-PCR Forward	CAATCTTCACATTACCACCTCCA
		qRT-PCR Reverse	CTCTAAAGAACATCCGTCAC
*MeCP2*	Total RNA	qRT-PCR Forward	TTCTATTCTGGGCTTTTGATTTGT
		qRT-PCR Reverse	CCCTTGTCCTACTCTATGGTTATCA
*GFAP*	Total RNA	qRT-PCR Forward	CGGAGACGCATCACCTCTG
		qRT-PCR Reverse	AGGGAGTGGAGGAGTCATTCG
*Tuj1*	Total RNA	qRT-PCR Forward	CAGCGATGAGCACGGCATAGAC
		qRT-PCR Reverse	CCAGGTTCCAAGTCCACCAGAATG
*Iba-1*	Total RNA	qRT-PCR Forward	ATCCCAAGTACAGCAGTGATGAGG
		qRT-PCR Reverse	AAATAGCTTTCTTGGCTGGGGGAC
*Syn1*	Total RNA	qRT-PCR Forward	CATTCTGGGATGGGCAAGGTCAAG
		qRT-PCR Reverse	GGCTCAGCAGTGGCATATGTCTTAG
*GAD67*	Total RNA	qRT-PCR Forward	TGCGCTTGGCTTTGGAA
		qRT-PCR Reverse	TCCCCCTTTCATTGCACTTT
*vGlut1*	Total RNA	qRT-PCR Forward	CTATGTCTAGCAGCTTCG
		qRT-PCR Reverse	TCAATGTATTTGCTCCT
*Olig2*	Total RNA	qRT-PCR Forward	CGGTGGCTTCAAGTCATCTTCCTC
		qRT-PCR Reverse	GGGCTCAGTCATCTGCTTCTTGTC
*Shank2*	Total RNA	qRT-PCR Forward	GAGCAGCCACCGTGATGATGAC
		qRT-PCR Reverse	ACCACCGTCTTGTCCTCAATAATGC
*Shank3*	Total RNA	qRT-PCR Forward	GATGTGCAAACCCGAGACTCTGAG
		qRT-PCR Reverse	TCCTCTGGTGACTTCCGCTCTTC
*miR-21a-3p*	miRNA	qRT-PCR Forward	CAACAGCAGTCGATGGGCT
*miR-18b-5p*	miRNA	qRT-PCR Forward	TAAGGTGCATCTAGTGCTGTTAG
*miR-10a-5p*	miRNA	qRT-PCR Forward	TACCCTGTAGATCCGAATTTGTG
*miR-155-5p*	miRNA	qRT-PCR Forward	TTAATGCTAATTGTGATAGGGGT
*miR-130b-5p*	miRNA	qRT-PCR Forward	ACTCTTTCCCTGTTGCACTACT
*let-7g-3p*	miRNA	qRT-PCR Forward	ACTGTACAGGCCACTGCCTTGC
*miR-218-2-3p*	miRNA	qRT-PCR Forward	CATGGTTCTGTCAAGCACCGCG
*miR-874-3p*	miRNA	qRT-PCR Forward	CTGCCCTGGCCCGAGGGACCGA
*miR-107-5p*	miRNA	qRT-PCR Forward	AGCTTCTTTACAGTGTTGCCTTG
*miR-129-2-3p*	miRNA	qRT-PCR Forward	AAGCCCTTACCCCAAAAAGCAT
*miR-425-3p*	miRNA	qRT-PCR Forward	ATCGGGAATGTCGTGTCCGCC
*miR-23a-3p*	miRNA	qRT-PCR Forward	ATCACATTGCCAGGGATTTCC
*miR-363-3p*	miRNA	qRT-PCR Forward	AATTGCACGGTATCCATCTGTA
*miR-491-5p*	miRNA	qRT-PCR Forward	AGTGGGGAACCCTTCCATGAGG
*miR-103-3p*	miRNA	qRT-PCR Forward	AGCAGCATTGTACAGGGCTATGA
*miR-199a-5p*	miRNA	qRT-PCR Forward	CCCAGTGTTCAGACTACCTGTTC
*U6*	miRNA	qRT-PCR Forward	CGCTTCGGCAGCACATATAC
*U6*	miRNA	qRT-PCR Reverse	AATTTGCGTGTCATCCTTGC
*mmu-Malat1*	LncRNA	qRT-PCR Forward	GCGAGCAGGCATTGTGGAGAG
		qRT-PCR Reverse	GCCGACCTCAAGGAATGTTACCG
*mmu-Dlx6os1*	LncRNA	qRT-PCR Forward	CTGAAGACTGACTGAGCGTGGAAG
		qRT-PCR Reverse	TCTGGGCGTAGGTTTCTCTCTGG
*mmu-Ifngas1*	LncRNA	qRT-PCR Forward	GGAGGCTAGTGTCTGGATGTTGTTG
		qRT-PCR Reverse	AGACTGGTGGCTGCTCTGAACTC
*mmu-Miat*	LncRNA	qRT-PCR Forward	TTTGCCTTTCTGGTCTGTTCCTTCC
		qRT-PCR Reverse	CCGCCATCATCCAAGCCGTTAG
*mmu-Snhg3*	LncRNA	qRT-PCR Forward	TCGCTCTCTTGGTGTGCTTGTTC
		qRT-PCR Reverse	CCCCGCTGATTCTCTTCTTTCCTC
*mmu-Epb41l4aos*	LncRNA	qRT-PCR Forward	GGGCGGGAATAAAGCGAAGACC
		qRT-PCR Reverse	GGACACCCTTTAACCCACTCTGTG
*mmu-TUG1*	LncRNA	qRT-PCR Forward	TTCCTACCACCTTACTACTGACG
		qRT-PCR Reverse	GGAGGTAAAGGCCACATC
*mmu-MEG3*	LncRNA	qRT-PCR Forward	TTGCAAGGGGCAAGGACTCTC
		qRT-PCR Reverse	TCATGTCGTCGGTTGGAAAGG
*mmu-NEAT1*	LncRNA	qRT-PCR Forward	GTTCCGTGCTTCCTCTTCTG
		qRT-PCR Reverse	CAGGGTGTCCTCCACCTTTA
*hsa-MALAT1*	LncRNA	qRT-PCR Forward	CCAGGTGCTACACAGAAGTGGAT
		qRT-PCR Reverse	GCTTGCTCGCTTGCTCCTCAG
*hsa-DLX6-AS1*	LncRNA	qRT-PCR Forward	TTCAAGCGATTCTCCTGCCTCAAG
		qRT-PCR Reverse	CAATGTGGCGAAACCCCGTCTC
*hsa-IFNG-AS1*	LncRNA	qRT-PCR Forward	AAGCCCACCAACTGCTAACAACC
		qRT-PCR Reverse	ACCCTTCAAAGACTTTCCCAACTGG
*hsa-MIAT*	LncRNA	qRT-PCR Forward	ACTAACTCCTGCCTTCCTGGTCTG
		qRT-PCR Reverse	CCAGCCATGCCGACATCCAAG
*hsa-SNHG3*	LncRNA	qRT-PCR Forward	TGCCTCAGCCTCCCAAGTAGC
		qRT-PCR Reverse	TGGGCGGATCACGAGGTCAG
*hsa-EPB41L4A-AS1*	LncRNA	qRT-PCR Forward	TCGGTCCCTCACTGGCACTTC
		qRT-PCR Reverse	CAGGCTTCCGTCCCACAATGC
*hsa-NEAT1*	LncRNA	qRT-PCR Forward	CCAGTGTGAGTCCTAGCATTGC
		qRT-PCR Reverse	CCTGGAAACAGAACATTGGAGAAC
*hsa-TUG1*	LncRNA	qRT-PCR Forward	ACCGGAGGAGCCATCTTGTC
		qRT-PCR Reverse	GAAAGAGCCGCCAACCGATC
*hsa-MEG3*	LncRNA	qRT-PCR Forward	TGGCATAGAGGAGGTGAT
		qRT-PCR Reverse	GGAGTGCTGTTGGAGAATA

### Western blot (WB)

WB was conducted following the methodology described in a previous publication [47]. The following antibodies were applied: Rabbit Anti-CD9 antibody (1:1000, Abcam, UK, ab92726), Rabbit Anti-CD63 antibody (1:1000, Abcam, UK, ab134045), Rabbit Anti-CD81 antibody (1:1000, Abcam, UK, ab109201), Rabbit Anti-Calnexin antibody (1:1000, Abcam, UK, ab22595), Rabbit Anti-Caspase-9 antibody (1:1000, Abcam, UK, ab22595), Rabbit Anti-Phospho-AKT(Ser473) antibody (1:1000, Absin, abs130002), Rabbit Anti-AKT antibody (1:1000, CST, Beverly, MA, USA, 4685S), Rabbit Anti-BAX antibody (1:8000, Proteintech, 50599-2-Ig), Mouse Anti-Bcl2 antibody (1:2000, Proteintech, 68103-1-Ig), Mouse Anti-GAPDH antibody (1:1000, Servicebio, GB15002). Using Odyssey Infrared Imaging System, the original images of the membranes were recorded and analyzed according to the ECL WB Protocol (Bio-Rad, Milan, Italy).

### Data analysis

The statistical analysis was performed using GraphPad Prism. In the figure legends, data were presented as the mean ± standard deviation (S.D.) or ± standard error of the mean (S.E.M.). A *p* value < 0.05 was considered statistically significant. Adobe Illustrator CC software and Figdraw (https://www.figdraw.com/static/index.html) were used to draw the chart for the specific mechanism of hADSC-Exo treatment of ASD.

### Supplementary Information

**Additional file 1: Fig. S1.** The exosomes of hADSC and hUCSC were extracted by ultrahigh speed gradient centrifugation. **Fig. S2.** lncRNA microarray data of hADSC-Exo and hUCSC-Exo. a Schematic diagram of lncRNA sampling. 2 hADSC-Exo simples and 3 hUCSC-Exo samples. b Sample correlation heat map. Correlations were performed using Pearsons correlation analysis. c Different lncRNA expression profiles among samples from the RNA sequencing data shown by heat map. d Wayne diagram of the differential proteins among the experimental samples. e–f Venn map showing the intersection of hADSC-Exo lncRNA and hUCMSC-Exo lncRNA. hUCMSC-Exo includes 729 lncRNAs, while hADSC-Exo includes 13,915 lncRNAs, encompassing all the lncRNAs found in hUCMSC-Exo. **Fig. S3.** Imaging of brain organoids after coculture with hADSCs-Exo. a Co-culture was treated with various does of hADSCs-Exo (40, 100 and 200 μM). Representative co-culture pictures are shown at Day1, Day12 and Day30. Scale bar = 500 μm. Organoid diameter after co-culture with the different doses of brain organoids at varied concentrations. b and c Immunofluorescence double-staining of formalin-fixed mice brain sections. b GFAP fluorescence in green and Tuj1 immunofluorescence in red (Texas Red), and DAPI staining (blue fluorescence). c Nestin immunofluorescence (green) and Ki-67 immunofluorescence (red) shown with DAPI (blue) stained nuclei. **Fig. S4.** BTBR mice were observed for the appearance of neurological signs. a The observational finding between BTBR mice and WT mice included epilation. A typical picture of BTBR mice with epilation symptoms at 6 months of age. b Adult BTBR mice also present higher weight compared to WT. c No abnormality was observed in other organs. d Abnormal synaptic growth and/or development and indirectly impair neurogenesis homeostasis in BTBR mice. **Fig. S5.** Representative pictures of behavior tests. a Representative pictures of novel object preference test (NOP). b Representative pictures of three-chambered test. **Fig. S6.** Firefly luciferase activity was normalized to Renilla luciferase activity (Firefly/Renilla). In 293T cells, Epb4114aos 3′-UTR pmirGLO plasmid with miR-21a-3p mimic/negative control or mutant Epb4114aos (pmirGLO-Ifngas1-MUT) 3′-UTR pmirGLO plasmid with miR-21a-3p mimic/negative control was co-transfected.

## Data Availability

The datasets used and/or analyzed during the current study are available from the corresponding author on reasonable request.
