# Supplementary material for: Exosome lncRNA IFNG-AS1 derived from mesenchymal stem cells of human adipose ameliorates neurogenesis and ASD-like behavior in BTBR mice
Source: J Nanobiotechnology. 2024 Feb 17;22:66. doi: 10.1186/s12951-024-02338-2 (PMC10874555; doi:10.1186/s12951-024-02338-2)
Supplement: Supplementary file 1 — Additional file 1: Fig. S1. The exosomes of hADSC and hUCSC were extracted by ultrahigh speed gradient centrifugation. Fig. S2. lncRNA microarray data of hADSC-Exo and hUCSC-Exo. a Schematic diagram of lncRNA sampling. 2 hADSC-Exo simples and 3 hUCSC-Exo samples. b Sample correlation heat map. Correlations were performed using Pearsons correlation analysis. c Different lncRNA expression profiles among samples from the RNA sequencing data shown by heat map. d Wayne diagram of the differential proteins among the experimental samples. e–f Venn map showing the intersection of hADSC-Exo lncRNA and hUCMSC-Exo lncRNA. hUCMSC-Exo includes 729 lncRNAs, while hADSC-Exo includes 13,915 lncRNAs, encompassing all the lncRNAs found in hUCMSC-Exo. Fig. S3. Imaging of brain organoids after coculture with hADSCs-Exo. a Co-culture was treated with various does of hADSCs-Exo (40, 100 and 200 μM). Representative co-culture pictures are shown at Day1, Day12 and Day30. Scale bar = 500 μm. Organoid diameter after co-culture with the different doses of brain organoids at varied concentrations. b and c Immunofluorescence double-staining of formalin-fixed mice brain sections. b GFAP fluorescence in green and Tuj1 immunofluorescence in red (Texas Red), and DAPI staining (blue fluorescence). c Nestin immunofluorescence (green) and Ki-67 immunofluorescence (red) shown with DAPI (blue) stained nuclei. Fig. S4. BTBR mice were observed for the appearance of neurological signs. a The observational finding between BTBR mice and WT mice included epilation. A typical picture of BTBR mice with epilation symptoms at 6 months of age. b Adult BTBR mice also present higher weight compared to WT. c No abnormality was observed in other organs. d Abnormal synaptic growth and/or development and indirectly impair neurogenesis homeostasis in BTBR mice. Fig. S5. Representative pictures of behavior tests. a Representative pictures of novel object preference test (NOP). b Representative pictures of three-chambered [file 12951_2024_2338_MOESM1_ESM.docx]

Additional figure


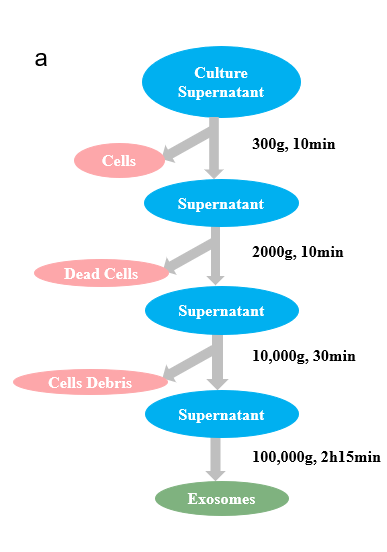


Fig. S1. The exosomes of hADSC and hUCSC were extracted by ultrahigh speed gradient centrifugation.


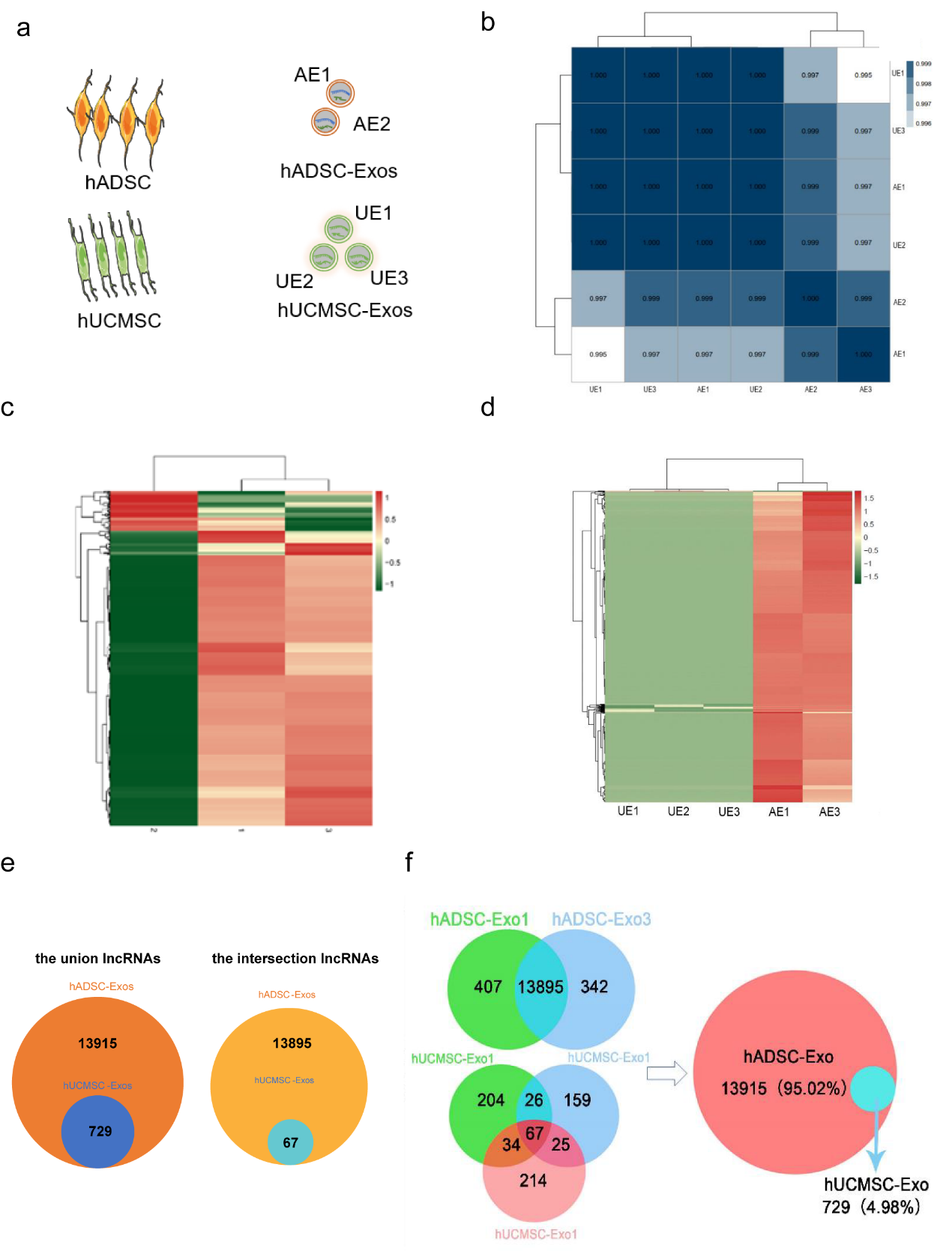


Fig. S2. lncRNA microarray data of hADSC-Exo and hUCSC-Exo. a Schematic diagram of lncRNA sampling. 2 hADSC-Exo simples and 3 hUCSC-Exo samples. b Sample correlation heat map. Correlations were performed using Pearsons correlation analysis. c Different lncRNA expression profiles among samples from the RNA sequencing data shown by heat map. d Wayne diagram of the differential proteins among the experimental samples. e-f Venn map showing the intersection of hADSC-Exo lncRNA and hUCMSC-Exo lncRNA. hUCMSC-Exo includes 729 lncRNAs, while hADSC-Exo includes 13,915 lncRNAs, encompassing all the lncRNAs found in hUCMSC-Exo.


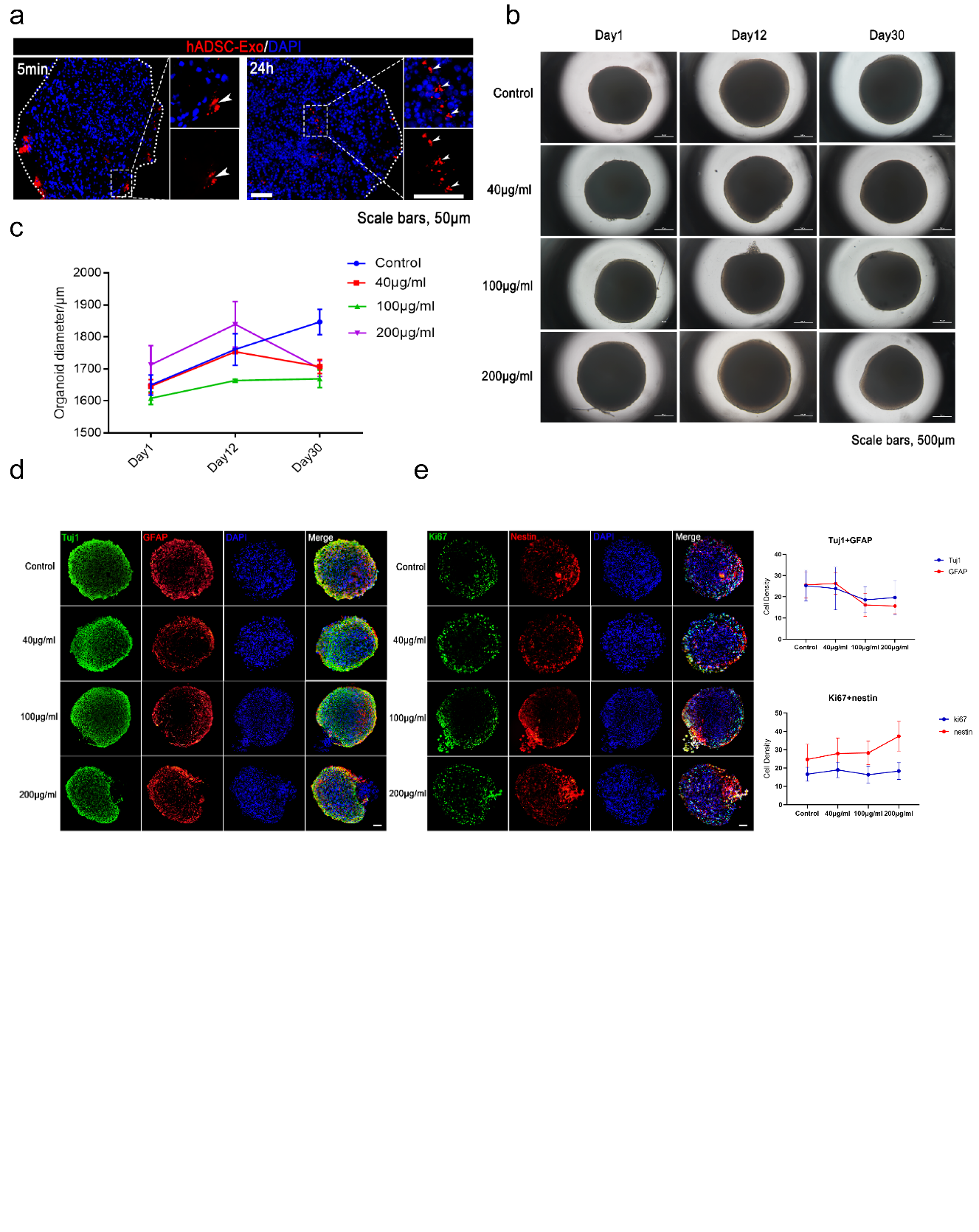


Fig. S3. Imaging of brain organoids after coculture with hADSCs-Exo. a Co-culture was treated with various does of hADSCs-Exo (40, 100 and 200 μM). Representative co-culture pictures are shown at Day1, Day12 and Day30. Scale bar=500 μm. Organoid diameter after co-culture with the different doses of brain organoids at varied concentrations. b and c Immunofluorescence double-staining of formalin-fixed mice brain sections. b GFAP fluorescence in green and Tuj1 immunofluorescence in red (Texas Red), and DAPI staining (blue fluorescence). c Nestin immunofluorescence (green) and Ki-67 immunofluorescence (red) shown with DAPI (blue) stained nuclei.


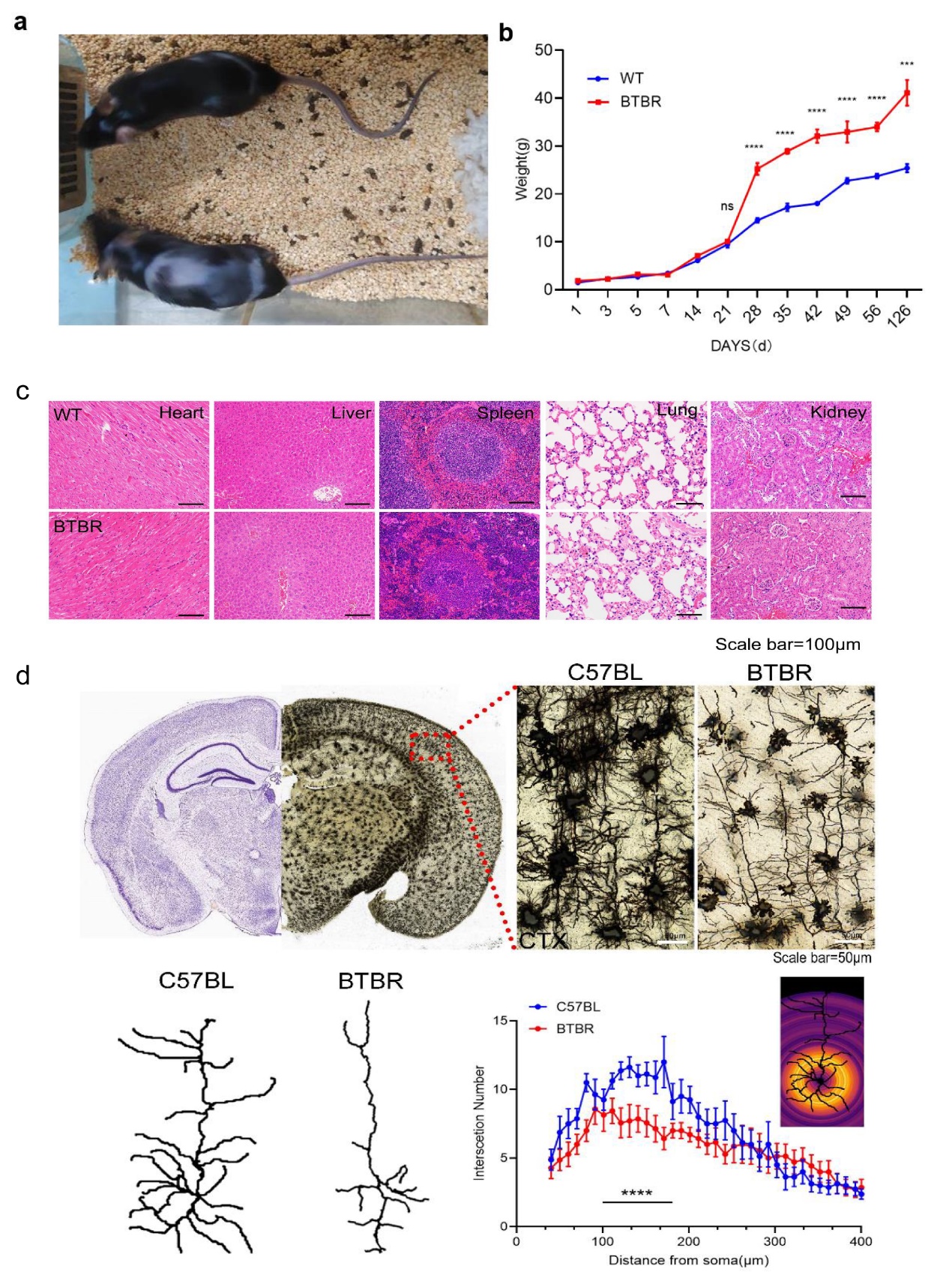


Fig. S4. BTBR mice were observed for the appearance of neurological signs. a The observational finding between BTBR mice and WT mice included epilation. A typical picture of BTBR mice with epilation symptoms at six months of age. b Adult BTBR mice also present higher weight compared to WT. c No abnormality was observed in other organs. d Abnormal synaptic growth and/or development and indirectly impair neurogenesis homeostasis in BTBR mice.


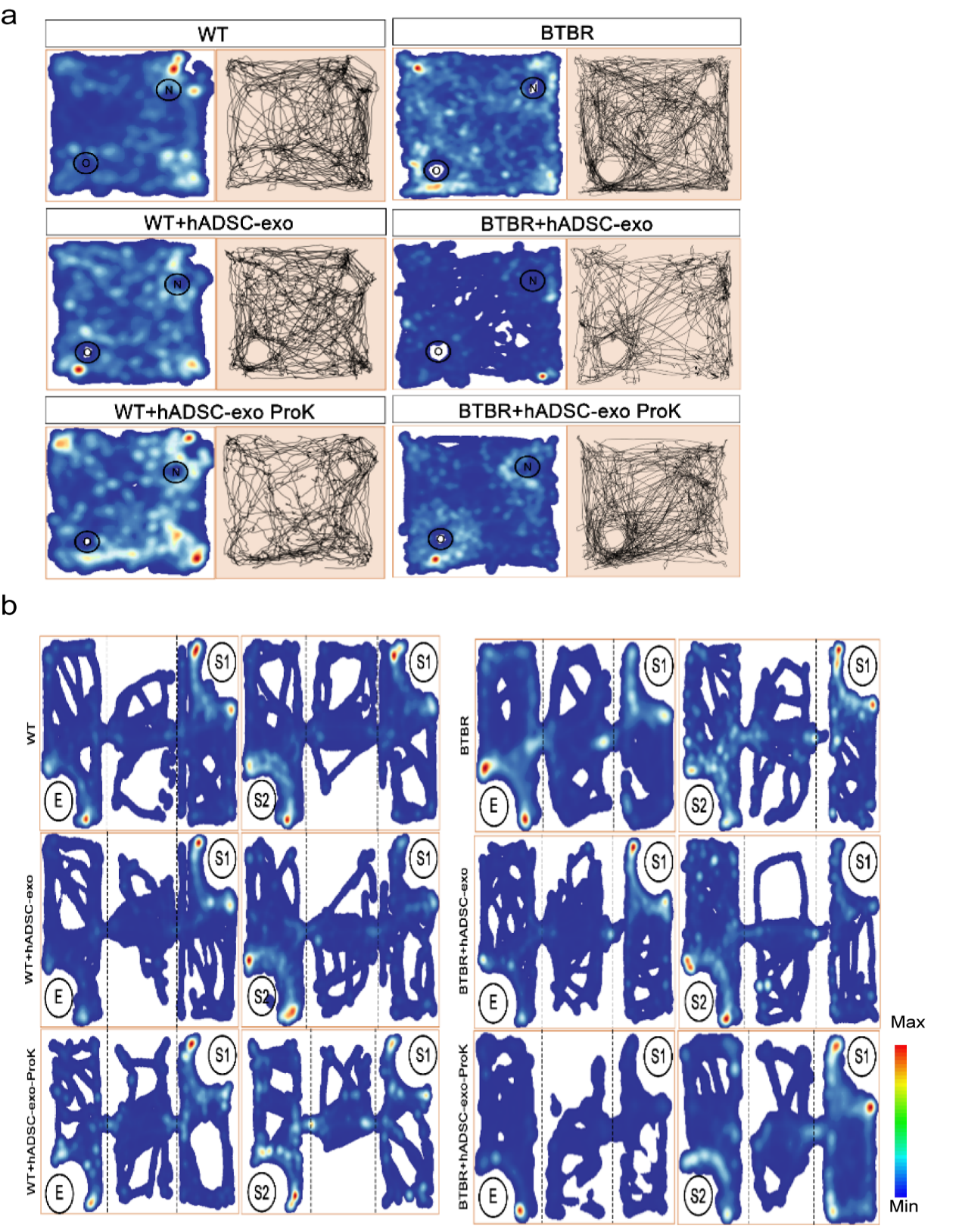


Fig. S5. Representative pictures of behavior tests. a Representative pictures of novel object preference test (NOP). b Representative pictures of three-chambered test.


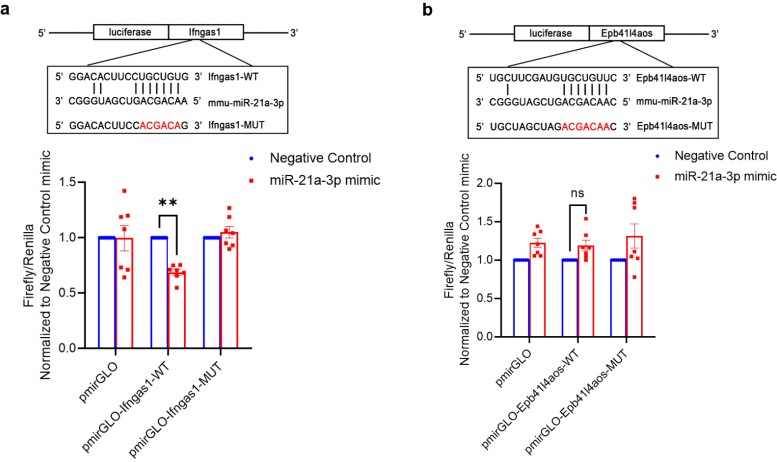


Fig. S6. Firefly luciferase activity was normalized to Renilla luciferase activity (Firefly/ Renilla). In 293T cells, Epb4114aos 3'-UTR pmirGLO plasmid with miR-21a-3p mimic/negative control or mutant Epb4114aos (pmirGLO-Ifngas1-MUT) 3'-UTR pmirGLO plasmid with miR-21a-3p mimic/negative control was co-transfected.
